# Model-Based Analysis of Low Stoichiometry Operation in Proton Exchange Membrane Water Electrolysis

**DOI:** 10.3390/membranes11090696

**Published:** 2021-09-09

**Authors:** Christoph Immerz, Boris Bensmann, Richard Hanke-Rauschenbach

**Affiliations:** Institute of Electric Power Systems, Leibniz Universität Hannover, 30167 Hannover, Germany; christoph.immerz@ifes.uni-hannover.de (C.I.); richard.hanke-rauschenbach@ifes.uni-hannover.de (R.H.-R.)

**Keywords:** 1+1-dimensional modeling, proton exchange membrane water electrolysis, current density distribution, low stoichiometry operation

## Abstract

Proton exchange membrane water electrolysis cells are typically operated with high water flow rates in order to guarantee the feed supply for the reaction, the hydration of the ionomer phase and to homogenize the temperature distribution. However, the influence of low flow rates on the cell behavior and the cell performance cannot be fully explained. In this work, we developed a simple 1+1-dimensional mathematical model to analyze the cell polarization, current density distribution and the water flow paths inside a cell under low stoichiometry condition. The model analysis is in strong context to previous experimental findings on low water stoichiometry operations. The presented analysis shows that the low water stoichiometry can lead to dry-out at the outlet region of the anode channel, while a water splitting reaction is also present there. The simulation results show that the supply with water in this region is achieved by a net water transport from the cathode to the anode catalyst layer resulting in higher local proton resistances in the membrane and the anode catalyst layer.

## 1. Introduction

Typically, proton exchange membrane water electrolysis (PEMWE) cells are operated with very high anode feed water flow rates. Such high flow rates guarantee a sufficient supply of the cells or stacks, as water serves as a reactant, hydration agent and cooling media [1,2], in particular, when heading towards current density ranges of $10\;A/cm^{2}$ [3]. Hence, there are necessities to optimize the water flow rate in order to maintain a compact system and control the thermal management of cells and stacks in particular.

Additionally, there is a need to fully understand the effects of two-phase flow on the electrochemical performance. By varying different operating conditions deeper insights in transport phenomena on a laboratory scale are gained, which have to be translated into the technicums or large demonstrator sizes to scale-up laboratory results into industrial orders of magnitude.

**Figure 1:**
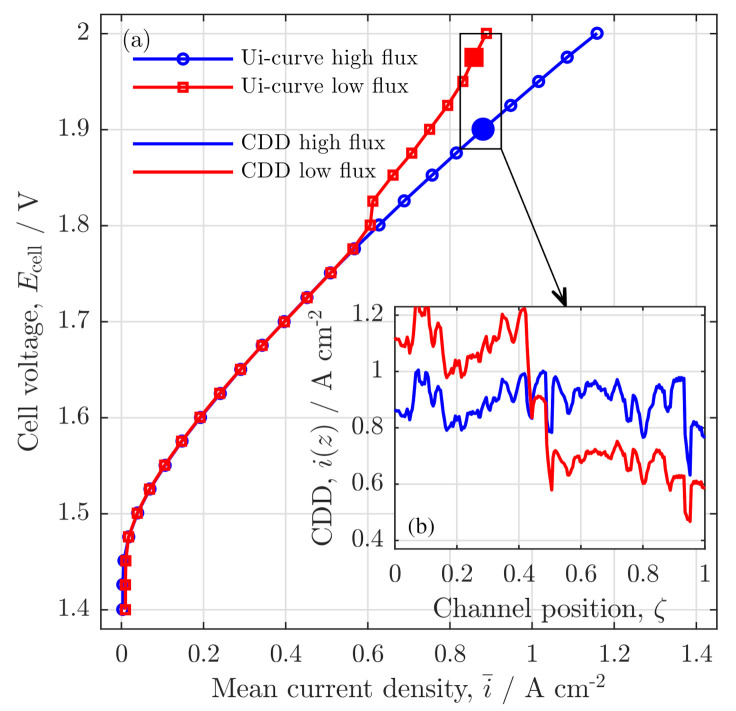
Experimental results from the analysis of high and low water stoichiometry operation on (**a**) the polarization curves and (**b**) the current density distribution (CDD). In (**a**): the blue line with circles shows results from experiments with a high water stoichiometry, the red line with squares shows the results from experiments, in which water ratios of $\lambda_{\mathrm{st}}\leq 5$ are reached for mean current densities of $\bar{i}\geq 0.6\;A/cm^{2}$. In (**b**): CDD for high (blue) and low (red line) stoichiometry operations at a common mean current density of $\bar{i}\leq 0.85\;A/cm^{2}$ are shown along the channel coordinate (ζ). The experimental setup is briefly described in the Appendix C. Data and comprehensive experiment description in Immerz et al. [5].

In the present study, we analyzed low water flow rates in PEMWE cells focusing on the influences of low stoichiometry on local cell behavior. For this purpose, a mathematical model is developed to describe and analyze the experimental findings that were observed in previous studies for a PEMWE cell of 50 cm channel length [4,5]. The main results from these experimental findings are summarized in Figure 1, showing the polarization curves (a) and current density distributions (CDD) (b) for high and low stoichiometry operations, which will be described with the herein developed model.

The previous modeling studies presented in the literature developed multidimensional models dealing with the optimization of two-phase flow in PEMWE cells. Typically, the models focused on the detailed description of the water flow rates and the associated two-phase flow in two- or three-dimensional models but did not couple these to the electrochemistry (e.g., [6,7]).

Based on those models, more-dimensional models were developed recently that contained a complex coupling of electrochemical processes, two-phase flow and the heat transfer in the cell (e.g., [8,9,10,11]). The focus of all these works was to describe and optimize the PEMWE at typical high water stoichiometry. In contrast, Onda et al. [12] established an early but extensive model, supported by experimental analyses, in order to investigate reduced water flow rates and low water stoichiometry operation. Although the model showed excellent agreement when validated with high water stoichiometry, the model was not able to represent the effects of a water starvation with low stoichiometry.

To the best of our knowledge, those low water stoichiometry conditions are not an investigated model thus far in the PEMWE literature. In this work, critically low water stoichiometric ratios are modeled and analyzed with a more-dimensional model. A particular focus is on (i) the description and enlightening of the experimental findings of previous works on low stoichiometry operation (e.g., [4,5,12,13,14]), (ii) the analysis of the water management in PEMWE in low stoichiometry operation and (iii) the assessment of the low stoichiometry operation mode in context of general use cases and thermal issues. As a starting point, the 1-dimensional sandwich model of Trinke [15] is used and reasonably simplified in order to describe the sandwich direction of a PEMWE cell and extended by a 1-dimensional channel model, that can describe local effects of the low stoichiometry operation.

In the following, a detailed analysis of the model results is given with a focus on the qualitative comprehension of experimental and model data. First, simulation results are investigated under both high and low stoichiometry operation modes and validated with the experimental findings from our previous work [5]. Furthermore, a deeper analysis of the local phenomena is provided, analyzing the local water flow rates in the channel and sandwich direction complemented by a local overpotential analysis under low stoichiometry operations. The model is used further to investigate operation parameters, including the temperature and pressure, with regard to the dehumidification behavior and further safety aspects. Finally, the low stoichiometry operation mode is critically analyzed.

## 2. Model Description

**Figure 2:**
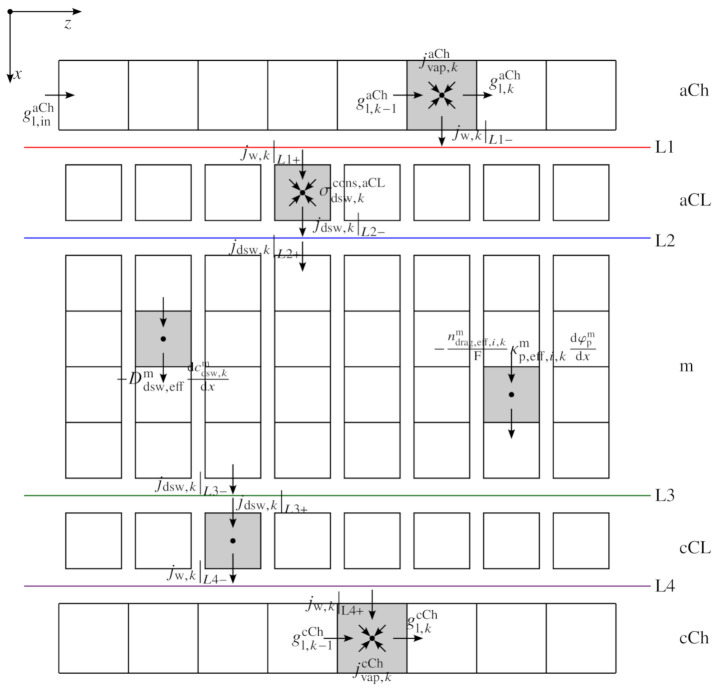
Schematic discretization pattern (exemplary for liquid and dissolved water): sandwich direction divided into the anode channel (aCh), anode catalyst layer (aCL), membrane (m), cathode catalyst layer (cCL) and the cathode channel (cCh). Boundaries between the layers L1, L2, L3 and L4. Channel water flux densities in the *z*-direction are represented as $g_{l,k}^{\mathrm{aCh}/\mathrm{cCh}}$, sandwich flux densities as $j_{\mathrm{dsw},k}$ for dissolved water and $j_{w,k}$ for liquid water. $\sigma_{\mathrm{dsw},k}^{\mathrm{cons},\mathrm{aCL}}$ represents the electrochemically consumed water sink term. A detailed description of the physical quantities is found in the model chapter.

The model is set up as a one-dimensional model in the channel coordinate (*z*) combined with a quasi one-dimensional model for the sandwich coordinate (*x*). In Figure 2 a schematic overview of the particular model discretization is given.

The models for the anode and cathode channels are cut off from the sandwich model along the boundaries L1 and L4. In between the boundaries L1 and L4, the sandwich model is built up, consisting of the anode catalyst layer, the membrane and the cathode catalyst layer. The sandwich model, which is based on Trinke’s model [15], is solved consecutively for each element *k* in the channel direction. The individual sandwich model paths are not connected directly in the *z*-axis (no in-plane transport). Instead, the coupling of the independent paths is achieved by the channel model enclosing all *k* sandwich model paths. The steady state model is implemented in *MATLAB* and numerically solved by the nonlinear system solver *fsolve*.

In the following, the sandwich model is described in detail, focusing on the simplifications in comparison to the model of Trinke [15] first. In a second step the channel model is presented, enabling a local analysis similar to the experimental results from Immerz et al. [5]. The experimental setup is briefly described in the Appendix C.

### 2.1. Sandwich Model

The sandwich model is based on the model of Trinke [15]. In context of the herein analysis the model is modified to couple with the superimposed channel model and is reasonably simplified in order to get a compact and robust system. First, the simplifications and assumptions in contrast to the model of Trinke [15] are described.

The present model is fully isothermal, leading to a model system that is built exclusively on mass and charge balances. The model is set up for the three layers: the anode catalyst layer (aCL), the membrane (m) and the cathode catalyst layer (cCL), while the porous transport layers (PTLs) are described in a simplified fashion (between boundaries L1 and L4 in Figure 2). Regarding the x-direction, both CLs are modeled as zero-dimensional and the membrane is discretized into five elements (n=5). In between the boundaries L1 and L4, only dissolved water flows in the ionomer phase, and dissolved hydrogen and oxygen in the ionomer’s water are considered.

The vapor phase is only considered for the water adsorption and desorption (across boundaries L1 and L4) but is not applied for the dissolved phase. Furthermore, a proton flux for the internal charge transport is considered, the electron flux is only determined for the catalyst layers. An electron leaking flux across the membrane (across L2 and L3) is neglected. The model does not consider a recombination of hydrogen and oxygen on either sides.

An overview on the model equations is given in Table 1. Furthermore, the Appendix A gives a summary of the system of equations. In the following, the most relevant equations are described in detail.

#### 2.1.1. Charge Balances

In the following, section, the determination of the electron potential is described first, followed by the proton potential. Finally, further necessary equations for the charge balance model are described.

##### Electron Potential

The electron potential is described by Ohm’s law for the anode and cathode catalyst layer.

(1) $$\begin{array}{cccc} & \varphi_{e,k}^{\mathrm{aCL}}: & \;0 & =-\left(0-i_{e,k}{|}_{L1^{+}}\right)+\delta^{\mathrm{aCL}}\sigma_{e,k}^{\mathrm{aCL}} \end{array}$$

(2) $$\begin{array}{cccc} & i_{e,k}{|}_{L1^{+}}: & \;i_{e,k}{|}_{L1^{+}} & =-\kappa_{e}^{\mathrm{aCL}}\frac{\varphi_{e,k}^{\mathrm{aCL}}-\varphi_{e,k}{|}_{L1}}{\delta^{\mathrm{aCL}}/2} \end{array}$$

(3) $$\begin{array}{cccc} & \varphi_{e,k}{|}_{L1}: & \;0 & =\varphi_{e,k}{|}_{L1}-\varphi_{e}^{a,\mathrm{set}} \end{array}$$

In Equations (1)–(3), the electron potential field in the aCL is described. Here, the electron flux density ${\left.i_{e,k}\right|}_{L1^{+}}$ crosses the imaginary interface between PTL and aCL, referred to as L1 in the following. The positive sign in the nomenclature always indicates a flux into a control element at a particular boundary, whereas a negative sign describes a flux out of an element. Furthermore, the electron flux is distributed along each channel coordinate, represented by the index *k* (s. also Figure 2).

The electron flux is driven by the potential difference between the electron potential on boundary L1, ${\left.\varphi_{e,k}\right|}_{L1}$ and the potential in the aCL, $\varphi_{e,k}^{\mathrm{aCL}}$, dependent on the electrical conductivity of the aCL $\kappa_{e}^{\mathrm{aCL}}$. Furthermore, in Equation (1), $\sigma_{e,k}^{\mathrm{aCL}}$ represents the electric source term (all source terms are given accumulated below in Equations (10) and (11)), $\delta^{\mathrm{aCL}}$ is the thickness of the aCL. The electron potential on the interface L1 is set (s. $\varphi_{e}^{a,\mathrm{set}}$ in Equation (3)) as a boundary condition, which is typically set to the cell voltage, $E_{\mathrm{cell}}$.

Since the electron flux across the membrane is neglected, no electron potential field is modeled in the membrane. The cathode side is modeled similarly (s. Table A1). Here, an electron current density is defined solely across the boundary L4, ${\left.i_{e,k}\right|}_{L4^{-}}$. The electron potential at interface L4, ${\left.\varphi_{e,k}\right|}_{L4}$, is typically set to zero, $\varphi_{e}^{c,\mathrm{set}}=0V$.

##### Proton Potential

The proton potential field is also described by Ohm’s law. For the anode side, the proton current on the interface L1 is set to zero, while the proton current density across the boundary L2, ${\left.i_{p,k}\right|}_{L2^{-}}$ is calculated from the proton source term, $\sigma_{p,k}^{\mathrm{aCL}}$ (s. Equation (10)).

(4) $$\begin{array}{cccc} & \varphi_{p,k}^{\mathrm{aCL}}: & \;0 & =-\left(i_{p,k}{|}_{L2^{-}}-0\right)+\delta^{\mathrm{aCL}}\sigma_{p,k}^{\mathrm{aCL}} \end{array}$$

(5) $$\begin{array}{cccc} & i_{p,k}{|}_{L2-}: & \;i_{p,k}{|}_{L2^{-}} & =-\kappa_{p,\mathrm{eff},k}^{\mathrm{aCL}}\frac{\varphi_{p,k}{|}_{L2}\varphi_{p,k}^{\mathrm{aCL}}}{\delta^{\mathrm{aCL}}/2} \end{array}$$

(6) $$\begin{array}{cccc} & \varphi_{p,k}{|}_{L2}: & \;0 & =i_{p,k}{|}_{L2^{-}}-i_{p,k}{|}_{L2^{+}} \end{array}$$

To determine the proton current across the boundary L2 in Equation (5), the proton potential in the aCL, $\varphi_{p,k}^{\mathrm{aCL}}$ and the proton potential on the boundary L2, ${\left.\varphi_{p},k\right|}_{L2}$ are used. $\kappa_{p,\mathrm{eff},k}^{\mathrm{aCL}}$ represents the effective proton conductivity of the aCL. The latter is defined spatially resolved for each channel element *k* in Equation (16). Additionally, a proton potential field in the membrane is simply modeled by Ohm’s law.

(7) $$\begin{array}{cccc} & \varphi_{p,k}^{m}: & \;0 & =-\frac{d}{dx}\left(-\kappa_{p,\mathrm{eff},i,k}^{m}\frac{d\varphi_{p,k}^{m}}{dx}\right) \end{array}$$

(8) $$\begin{array}{cccc} & i_{p,k}{|}_{L2^{+}}: & \;i_{p,k}{|}_{L2^{+}} & =-\kappa_{p,\mathrm{eff},1,k}^{m}\frac{\varphi_{p,1,k}^{m}-\varphi_{p,k}{|}_{L2}}{\Delta x^{m}/2} \end{array}$$

(9) $$\begin{array}{cccc} & i_{p,k}{|}_{L3^{-}}: & \;i_{p,k}{|}_{L3^{-}} & =-\kappa_{p,\mathrm{eff},n,k}^{m}\frac{\varphi_{p,k}{|}_{L3}-\varphi_{p,n,k}^{m}}{\Delta x^{m}/2} \end{array}$$

Equations (7)–(9) contain the proton conductivity of the membrane, $\kappa_{p,\mathrm{eff},i,k}^{m}$, as function of the dissolved water content of the membrane (s. Equation (16)). The proton conductivity of the membrane is distributed in sandwich direction (s. index i=1,⋯,n−1) and along the channel coordinate (s. index k=1,⋯,m). Furthermore, $\Delta x^{m}$ represents the length of each control element, which is the thickness of the membrane, $\delta^{m}$, divided by the number of discrete membrane elements, *n* ($\Delta x^{m}=\delta^{m}/n$). The proton flux densities into and out of the membrane on boundaries L2 and L3 (${\left.i_{p,k}\right|}_{L2^{+}}$, ${\left.i_{p,k}\right|}_{L3^{-}}$ in Equations (8) and (9)) are calculated in the first (i=1) and the last (i=n) membrane control element.

The cCL proton potential is modeled analogously to the previously described aCL and is presented in Equations (A23)–(A25).

##### Further Equations to Solve the Charge Balances

Finally, further necessary equations are described in the following, starting with the source terms. The proton and electron source terms for the anode and the cathode side, $\sigma_{e/p,k}^{\mathrm{aCL}/\mathrm{cCL}}$, are calculated with Butler–Volmer approaches:

(10) $$\begin{array}{cc} \sigma_{e/p,k}^{\mathrm{aCL}} & =i_{0}^{\mathrm{aCL}}\;\cdot \;a_{\mathrm{cat}}^{\mathrm{aCL}}\left[\exp\left(\frac{\alpha_{\mathrm{ox}}^{\mathrm{aCL}}F}{RT}\;\cdot \;\eta_{\mathrm{act},k}^{\mathrm{aCL}}\right)-\exp\left(\frac{-\alpha_{\mathrm{red}}^{\mathrm{aCL}}F}{RT}\;\cdot \;\eta_{\mathrm{act},k}^{\mathrm{aCL}}\right)\right] \end{array}$$

(11) $$\begin{array}{cc} \sigma_{e/p,k}^{\mathrm{cCL}} & =i_{0}^{\mathrm{cCL}}\;\cdot \;a_{\mathrm{cat}}^{\mathrm{cCL}}\left[\exp\left(\frac{\alpha_{\mathrm{ox}}^{\mathrm{cCL}}F}{RT}\;\cdot \;\eta_{\mathrm{act},k}^{\mathrm{cCL}}\right)-\exp\left(\frac{-\alpha_{\mathrm{red}}^{\mathrm{cCL}}F}{RT}\;\cdot \;\eta_{\mathrm{act},k}^{\mathrm{cCL}}\right)\right] \end{array}$$

Herein, $i_{0}^{\mathrm{aCL}/\mathrm{cCL}}$ represent the exchange current densities, and $a_{\mathrm{cat}}^{\mathrm{aCL}/\mathrm{cCL}}$ represent the volume specific catalyst surfaces on the anode respectively cathode side. F is the Faraday’s constant, R is the universal gas constant, and $\alpha_{\mathrm{ox}/\mathrm{red}}^{\mathrm{aCL}/\mathrm{cCL}}$ represent the charge transfer coefficients. Furthermore, $\eta_{\mathrm{act},k}^{\mathrm{aCL}/\mathrm{cCL}}$ are the activation overpotentials on the anode and cathode, defined in Equations (12) and (13).

(12) $$\begin{array}{cc} \eta_{\mathrm{act},k}^{\mathrm{aCL}} & =\varphi_{e,k}^{\mathrm{aCL}}-\varphi_{p,k}^{\mathrm{aCL}}-E_{\mathrm{Nernst},k}^{\mathrm{aCL}} \end{array}$$

(13) $$\begin{array}{cc} \eta_{\mathrm{act},k}^{\mathrm{cCL}} & =\varphi_{e,k}^{\mathrm{cCL}}-\varphi_{p,k}^{\mathrm{cCL}}-E_{\mathrm{Nernst},k}^{\mathrm{cCL}} \end{array}$$

The activation overpotential is the difference between the proton and the electron potential of each half cell corrected by the Nernst potentials $E_{\mathrm{Nernst},k}^{\mathrm{aCL}/\mathrm{cCL}}$.

(14) $$\begin{array}{cc} E_{\mathrm{Nernst},k}^{\mathrm{aCL}} & =E_{0}^{\mathrm{aCL}}+\frac{RT}{2F}\;\cdot \;\ln\left(\frac{c_{\mathrm{dsw}}^{\mathrm{sat},l}}{c_{\mathrm{dsw},k}^{\mathrm{aCL}}}\;\cdot \;\sqrt{\frac{c_{\mathrm{dsg},O2,k}^{\mathrm{aCL}}}{c_{\mathrm{dsg},O2}^{0}}}\right) \end{array}$$

(15) $$\begin{array}{cc} E_{\mathrm{Nernst},k}^{\mathrm{cCL}} & =0\;V+\frac{RT}{2F}\;\cdot \;\ln\left(\frac{c_{\mathrm{dsg},H2,k}^{\mathrm{cCL}}}{c_{\mathrm{dsg},H2}^{0}}\right) \end{array}$$

In Equation (14), the Nernst potential of the anode side is dependent on the dissolved water concentration as educt, $c_{\mathrm{dsw},k}^{\mathrm{aCL}}$ (s. Equations (27)–(29)) and the dissolved oxygen concentration as product, $c_{\mathrm{dsg},O2,k}^{\mathrm{aCL}}$ (s. Equations (17)–(19)). On the cathode side, only the dissolved hydrogen concentration as product is taken into account $c_{\mathrm{dsg},H2,k}^{\mathrm{cCL}}$ (s. Equations (A26)–(A28)). In Equations (14) and (15) $c_{\mathrm{dsw}}^{\mathrm{sat},l}$, $c_{\mathrm{dsg},O2}^{0}$ and $c_{\mathrm{dsg},H2}^{0}$ represent the reference concentrations of liquid water, oxygen and hydrogen, respectively, given in Table A3 and Equation (A9). For the anode half cell, a temperature correction of the reference Nernst potential (s. $E_{0}^{\mathrm{aCL}}$) is shown in Equation (A1), while the cathode reference Nernst potential is defined as 0 V [16].

Finally, the effective proton conductivity, $\kappa_{p,\mathrm{eff},i,k}^{v}$ based on Springer et al. [17], is described as a function of temperature and the dissolved water content of each layer $\lambda_{i,k}^{v}$ (v=aCL,m,cCL).

(16) $$\kappa_{p,\mathrm{eff},i,k}^{v}=\frac{\epsilon_{\mathrm{ion}}^{v}}{\tau_{\mathrm{ion}}^{v}}\;\cdot \;\left(0.5139\;\cdot \;\lambda_{i,k}^{v}-0.326\right)\exp\left(1268\;\cdot \;\left(\frac{1}{303}-\frac{1}{T/K}\right)\right)$$

Since the catalyst layers are not spatially resolved in the sandwich direction, consequently the index *i* is ignored here. The porous character of the Nafion^®^ layers is taken into account by the Bruggemann approach [18], for which $\epsilon_{\mathrm{ion}}^{v}$ represents the volume fraction of the ionomer, and $\tau_{\mathrm{ion}}^{v}$ represents its tortuosity in each layer *v*. All further equations for the electrical model are given in Appendix A.

#### 2.1.2. Dissolved Gases

The dissolved gas concentrations of hydrogen and oxygen in the sandwich coordinate are considered in the aCL, the membrane and the cCL (v: aCL, m, cCL). First, the balance equations for the aCL are presented for each species *j* (j: H_2_, O_2_).

(17) $$\begin{array}{cccc} & c_{\mathrm{dsg},j,k}^{\mathrm{aCL}}: & \;0= & -\left(j_{\mathrm{dsg},j,k}{|}_{L2^{-}}-0\right)+\delta^{\mathrm{aCL}}\sigma_{\mathrm{dsg},j,k}^{\mathrm{evo},\mathrm{aCL}}-j_{j,k}{|}_{L1_{+}} \end{array}$$

(18) $$\begin{array}{cccc} & j_{\mathrm{dsg},j,k}{|}_{L2^{-}}: & \;j_{\mathrm{dsg},j,k}{|}_{L2^{-}} & =-D_{\mathrm{dsg},\mathrm{eff},j}^{\mathrm{aCL}}\frac{C_{\mathrm{dsg},j,k}{|}_{L2}-c_{\mathrm{dsg},j,k}^{\mathrm{aCL}}}{\delta^{\mathrm{aCL}}/2} \end{array}$$

(19) $$\begin{array}{cccc} & C_{\mathrm{dsg},j,k}{|}_{L2}: & \;0= & j_{\mathrm{dsg},j,k}{|}_{L2^{-}}-j_{\mathrm{dsg},j,k}{|}_{L2^{+}} \end{array}$$

In Equation (17), the evolved gases are determined with the source term $\sigma_{\mathrm{dsg},j,k}^{\mathrm{evo},\mathrm{aCL}}$. The source term is zero for hydrogen in the aCL and vice versa for oxygen in the cathode, since recombination is neglected in the model. The dissolved gases either desorb into the gaseous phase and leave the aCL across the boundary L1, ${\left.j_{j,k}\right|}_{L1+}$, or dissolved fluxes permeate through the membrane as crossover fluxes across boundary L2, ${\left.j_{\mathrm{dsg},j,k}\right|}_{L2^{-}}$. Pure diffusive transport with the effective diffusivity $D_{\mathrm{dsg},\mathrm{eff},j}^{\mathrm{aCL}}$ (s. Equation (A3)) is assumed for the dissolved gas transport in the anode, driven by the gradient of the layer concentration, $c_{\mathrm{dsg},j,k}^{\mathrm{aCL}}$ and the concentration on the boundary L2, ${\left.c_{\mathrm{dsg},j,k}\right|}_{L2}$.

The membrane model for dissolved gases (s. Equations (20)–(22)) is set up as simple diffusive transport model:

(20) $$\begin{array}{cccc} & c_{\mathrm{dsg},j,k}^{m}: & \;0 & =-\frac{d}{dx}\left(-D_{\mathrm{dsg},\mathrm{eff},j}^{m}\frac{dc_{\mathrm{dsg},j,k}^{m}}{dx}\right) \end{array}$$

(21) $$\begin{array}{cccc} & j_{\mathrm{dsg},j,k}{|}_{L2^{+}}: & \;j_{\mathrm{dsg},j,k}{|}_{L2^{+}} & =-D_{\mathrm{dsg},\mathrm{eff},j}^{m}\frac{c_{\mathrm{dsg},j,1,k}^{m}-C_{\mathrm{dsg},j,k}{|}_{L2}}{\Delta x^{m}/2} \end{array}$$

(22) $$\begin{array}{cccc} & j_{\mathrm{dsg},j,k}{|}_{L3^{-}}: & \;j_{\mathrm{dsg},j,k}{|}_{L3^{-}} & =-D_{\mathrm{dsg},\mathrm{eff},j}^{m}\frac{C_{\mathrm{dsg},j,k}{|}_{L3}-c_{\mathrm{dsg},j,n,k}^{m}}{\Delta x^{m}/2} \end{array}$$

In Equations (20)–(22), $D_{\mathrm{dsg},\mathrm{eff},j}^{m}$ represents the diffusion coefficient of each species *j*, defined in Equation (A3). The indexation of the dissolved gases (and the dissolved water model) is identical to the electrical model for the proton flux membrane model (s. previous section). The cathode catalyst layer is described analogously to the aCL, but vice versa for each species (s. Table A1, Equations (A26)–(A28)).

##### Further Equations

The source terms are given by Faraday’s law in Equation (23) for oxygen in the anode and in Equation () for hydrogen in the cathode.

(23) $$\begin{array}{cc} \sigma_{\mathrm{dsg},O2,k}^{\mathrm{evo}},\mathrm{aCL} & =\frac{\sigma_{p,k}^{\mathrm{aCL}}}{4F} \end{array}$$

(24) $$\begin{array}{cc} \sigma_{\mathrm{dsg},H2,k}^{\mathrm{evo}},\mathrm{cCL} & =-\frac{\sigma_{p,k}^{\mathrm{cCL}}}{2F} \end{array}$$

The gaseous fluxes into the channels across boundary L1 (${\left.j_{j,k}\right|}_{L1+}$) are described by the sorption dynamics from the dissolved form into the gaseous form of each species.

(25) $$\begin{array}{cc} j_{j,k}{|}_{L1+} & =\delta^{\mathrm{aCL}}k_{l,j}a_{\mathrm{ion}}\left(c_{\mathrm{dsg},j,k}^{\mathrm{aCL}}-c_{\mathrm{dsg},j}^{\mathrm{sat}}\right) \end{array}$$

(26) $$\begin{array}{cc} j_{j,k}{|}_{L4+} & =\delta^{\mathrm{cCL}}k_{l,j}a_{\mathrm{ion}}\left(c_{\mathrm{dsg},j,k}^{\mathrm{cCL}}-c_{\mathrm{dsg},j}^{\mathrm{sat}}\right) \end{array}$$

In Equations (25) and (26), $k_{l,j}$ is the mass transfer coefficient, $a_{\mathrm{ion}}$ is the volume specific ionomer surface of the catalyst layer, and $c_{\mathrm{dsg},j}^{\mathrm{sat}}$ is the saturation concentration of hydrogen and oxygen in water. As described earlier, it is assumed that only dissolved species are present in the catalyst layers. The species are adsorbed or desorbed directly at the interface. All parameter values are given in Table A3, respectively in Equations (A4) and (A5).

#### 2.1.3. Dissolved Water Model

In the dissolved water model, solely dissolved water fluxes are assumed to cross the internal MEA boundaries (e.g., ${\left.j_{\mathrm{dsw},k}\right|}_{L2^{-}}$). Only across boundary L1, liquid flux densities are considered as ${\left.j_{w,k}\right|}_{L1+}$.


(27) $$\begin{array}{ccc} c_{\mathrm{dsw},k}^{\mathrm{aCL}}: & 0= & -\left({\left.j_{\mathrm{dsw},k}\right|}_{L2^{-}}-0\right)+\delta^{\mathrm{aCL}}\sigma_{\mathrm{dsw},k}^{\mathrm{cons},\mathrm{aCL}}-j_{w,k}{|}_{L1+} \end{array}$$

(28) $$\begin{array}{ccc} j_{\mathrm{dsw},k}{|}_{L2^{-}}: & j_{\mathrm{dsw},k}{|}_{L2^{-}}= & -D_{\mathrm{dsw},\mathrm{eff}}^{\mathrm{aCL}}\frac{C_{\mathrm{dsw},k}{|}_{L2}-c_{\mathrm{dsw},k}^{\mathrm{aCL}}}{\delta^{\mathrm{aCL}}/2}-\frac{n_{\mathrm{drag},\mathrm{eff},k}^{\mathrm{aCL}}}{F}\kappa_{p,\mathrm{eff},k}^{\mathrm{aCL}}\frac{\varphi_{p,k}{|}_{L2}-\varphi_{p,k}^{\mathrm{aCL}}}{\delta^{\mathrm{aCL}}/2} \end{array}$$

(29) $$\begin{array}{ccc} c_{\mathrm{dsw},k}{|}_{L2}: & 0= & j_{\mathrm{dsw},k}{|}_{L2^{-}}-j_{\mathrm{dsw},k}{|}_{L2^{+}} \end{array}$$

In Equation (27), the source term $\sigma_{\mathrm{dsw},k}^{\mathrm{cons},\mathrm{aCL}}$ describes the amount of electrochemically consumed water, which is simply described by Faraday’s law.

(30) $$\sigma_{\mathrm{dsw},k}^{\mathrm{cons}},\mathrm{aCL}=\frac{\sigma_{p,k}^{\mathrm{aCL}}}{2F}$$

The dissolved water flux density (s. Equation (28)) is modeled as the sum of a diffusion flux density depending on the diffusivity $D_{\mathrm{dsw},\mathrm{eff}}^{\mathrm{aCL}}$ (s. Equation (A8)) and an electro-osmotic drag flux density dependent on the effective drag coefficient $n_{\mathrm{drag},\mathrm{eff},k}^{\mathrm{aCL}}$ (s. Equation (33)). The balance equations for the membrane and the catalyst layer are described in Table A1. The liquid water flux densities across L1 and L4 are described by the sorption dynamics.

(31) $$\begin{array}{cc} j_{w,k}{|}_{L1+} & =\delta^{\mathrm{aCL}}a_{\mathrm{ion}}\left(\left(1-\omega_{l,k}^{\mathrm{aCL}}\right)k_{\mathrm{vap}}^{\mathrm{sorp}}\left(c_{\mathrm{dsw},k}^{\mathrm{aCL}}-c_{\mathrm{dsg},\mathrm{vap},k}^{\mathrm{aCL}}\right)+\omega_{l,k}^{\mathrm{aCL}}k_{l}^{\mathrm{sorp}}\left(c_{\mathrm{dsw},k}^{\mathrm{aCL}}-c_{\mathrm{dsw}}^{\mathrm{sat}}\right)\right) \end{array}$$

(32) $$\begin{array}{cc} j_{w,k}{|}_{L4-} & =\delta^{\mathrm{aCL}}a_{\mathrm{ion}}\left(\left(1-\omega_{l,k}^{\mathrm{cCL}}\right)k_{\mathrm{vap}}^{\mathrm{sorp}}\left(c_{\mathrm{dsw},k}^{\mathrm{cCL}}-c_{\mathrm{dsg},\mathrm{vap},k}^{\mathrm{cCL}}\right)+\omega_{l,k}^{\mathrm{cCL}}k_{l}^{\mathrm{sorp}}\left(c_{\mathrm{dsw},k}^{\mathrm{cCL}}-c_{\mathrm{dsw}}^{\mathrm{sat}}\right)\right) \end{array}$$

In Equations (31) and ((32)), $k_{l}^{\mathrm{sorp}}$ represent the sorption coefficient from the liquid water phase across the boundary L1, and $k_{g}^{\mathrm{sorp}}$ is the sorption coefficient from the gaseous phase. The sorption dynamics are supplemented by the liquid water ratio, which is in contact with the ionomer surface in the catalyst layer $\omega_{l,k}^{\mathrm{aCL}/\mathrm{cCL}}$ (s. Equation (40)). It is assumed that only the part of the specific surface covered with water participates noticeably in the water sorption.

The differences between the dissolved water concentrations in the catalyst layer $c_{\mathrm{dsw},k}^{\mathrm{aCL}/\mathrm{cCL}}$ (s. Equation (27)) and the liquid saturation concentration $c_{\mathrm{dsw}}^{\mathrm{sat}}$ (based on Equation (A9) for $\lambda {{}^{\mathrm{sat}}}_{\mathrm{dsw}}=22$) act as the driving force for sorption from or into the liquid phase. For the sorption form or into the gaseous phase, the dissolved vapor concentrations in the catalyst layers, $c_{\mathrm{dsg},\mathrm{vap},k}^{\mathrm{aCL}/\mathrm{cCL}}$, are used as references (s. Equation (A14)).

Finally, the effective drag coefficient is described. In this model, it is calculated by an empirical function [12,15,17] dependent on the dissolved water concentration of each layer and the temperature.

(33) $$n_{\mathrm{drag},\mathrm{eff},k}^{v}=0.0134\;\cdot \;T\;\cdot \;\frac{c_{\mathrm{dsw},k}^{v}}{c_{\mathrm{sat},l}^{\mathrm{dsw}}}\;v:\mathrm{aCL},m,\mathrm{cCL}$$

### 2.2. Channel Model

In contrast to the model of Trinke [15] a channel model is considered to describe also along-the-channel phenomena. Balance equations are applied for liquid water, hydrogen, oxygen and vapor in the anode and the cathode channel. Due to the similarity between the anode and cathode model, only the anode model is presented here. Relevant equations for the cathode model are summarized in Table A2.

(34) $$g_{l,k}^{\mathrm{aCh}}:\;0=g_{l,k-1}^{\mathrm{aCh}}-g_{l,k}^{\mathrm{aCh}}+\frac{{\left.j_{w,k}\right|}_{L}1-}{\delta^{\mathrm{aCh}}}\;\cdot \;\Delta z-\frac{j_{\mathrm{vap},k}^{\mathrm{aCh}}}{\delta^{\mathrm{aCh}}}\;\cdot \;\Delta z$$

The liquid water balance in the anode channel (s. Equation (34)) accounts for the flux density into ($g_{l,k-1}^{\mathrm{aCh}}$) and out of ($g_{l,k}^{\mathrm{aCh}}$) each control element in the *z*-direction and the flux density towards (resp. from) the aCL (${\left.j_{w,k}\right|}_{L1-}$). A vapor flux density is considered as $j_{\mathrm{vap},k}^{\mathrm{aCh}}$. The gaseous product flux densities (s. Equation (35), with j: H_2_, O_2_) and the vapor flux density (s. Equation (36)) are similarly balanced.

(35) $$\begin{array}{cccc} & g_{j,k}^{\mathrm{aCh}}:\; & \;0= & g_{j,k-1}^{\mathrm{aCh}}-g_{j,k}^{\mathrm{aCh}}+\frac{{\left.j_{j,k}\right|}_{L1-}}{\delta^{\mathrm{aCh}}}\;\cdot \;\Delta z \end{array}$$

(36) $$\begin{array}{cccc} & g_{\mathrm{vap},k}^{\mathrm{aCh}}:\; & \;0= & g_{\mathrm{vap},k-1}^{\mathrm{aCh}}-g_{\mathrm{vap},k}^{\mathrm{aCh}}+\frac{j_{\mathrm{vap},k}^{\mathrm{aCh}}}{\delta^{\mathrm{aCh}}}\;\cdot \;\Delta z \end{array}$$

The flux densities from the channels into the PTLs are three-times higher than the flux densities from the PTLs to the CLs, since the width of the active area is three times the channel’s width (${\left.j_{w,k}\right|}_{L1+}=3\;\cdot \;{\left.j_{w,k}\right|}_{L1-}$).

For the vaporization, it is assumed that water vaporizes at the L1 and L4 boundaries and humidifies the evolving gas phase there. A simple vaporization kinetic is applied

(37) $$j_{\mathrm{vap},k}^{\mathrm{aCL}}=\delta^{\mathrm{aCL}}\;\cdot \;\frac{k^{\mathrm{vap}}a_{\mathrm{pore}}^{\mathrm{aCL}}}{RT}\left(p_{\mathrm{vap}}^{\mathrm{sat}}-p_{\mathrm{vap},k}^{\mathrm{aCh}}\right)$$

in which $k^{\mathrm{vap}}$ represents the vaporization rate, and $a_{\mathrm{pore}}^{\mathrm{aCL}}$ represents the volume-specific pore surface. The pressure difference between the anode channel partial pressure of vapor $p_{\mathrm{vap},k}^{\mathrm{aCh}}$ (s. Equation (A12)) and the vapor saturation pressure $p_{\mathrm{vap}}^{\mathrm{sat}}$ (s. Equation (A11)) acts as the driving force.

Based on the channel model, the water stoichiometry $\lambda_{\mathrm{st}}$ can be calculated as a fraction of the supplied water flux ($g_{l},{\mathrm{in}}^{\mathrm{aCh}}$) and the consumed water flux based on the Faraday’s law ($\sum_{1}^{k}\sigma_{\mathrm{dsw},k}^{\mathrm{cons},\mathrm{aCL}}\;\cdot \;\delta \mathrm{aCL}$), which can be easily expressed as

(38) $$\lambda_{\mathrm{st}}=\frac{{\dot{m}}_{\mathrm{in}}^{\mathrm{aCh}}}{{\tilde{M}}_{w}}\;\cdot \;\frac{2F}{\bar{i}\;\cdot \;A_{\mathrm{geo}}}$$

where ${\tilde{M}}_{w}$ is the molar mass of water, ${\dot{m}}_{\mathrm{in}}^{\mathrm{aCh}}$ is the feed water flow rate into the anode channel in $g\min^{-1}$, $\bar{i}$ is the mean current density of the cell, and $A_{\mathrm{geo}}$ is the geometric cell area.

### 2.3. Coupling of Channel and Sandwich Model

The model is strongly adapted to the experimental setup and data of our previous work [5]. The perspective of the model is the description of the experimental findings and a deeper analysis of the low stoichiometry operation mode. Here, reasonable simplifying assumptions are made to couple the channel and the sandwich model, without an in-depth description of the porous transport layer. Typically, the PTL conducts the two-phase flow of water and gases, which is strongly dependent on the interaction of its physical properties, including the hydrophilicity, porosity, pore diameter etc. Furthermore, the interfacial processes between the channel, PTL and CL are very complex and are still the objectives of recent scientific research (e.g., [19,20]).

To connect the conditions of the channel model with the sandwich model in the catalysts, a coupling between the volume ratio of liquid water in the channel, $\omega_{l,k}^{\mathrm{aCh}}$, and the liquid water ratio on the catalyst interface, $\omega_{l,k}^{\mathrm{aCL}}$, is established. First, the channel liquid ratio is calculated as

(39) $$\omega_{l,k}^{\mathrm{aCh}}=\frac{v_{l,k}^{\mathrm{aCh}}}{v_{l,k}^{\mathrm{aCh}}+v_{O_{2},k}^{\mathrm{aCh}}+v_{H_{2},k}^{\mathrm{aCh}}+v_{\mathrm{vap},k}^{\mathrm{aCh}}}$$

in which the volume flux densities (e.g., $v_{l,k}^{\mathrm{aCh}}$) are calculated from the molar fluxes with the ideal gas and ideal liquid law (s. Equations (A16)–(A19)).

**Figure 3:**
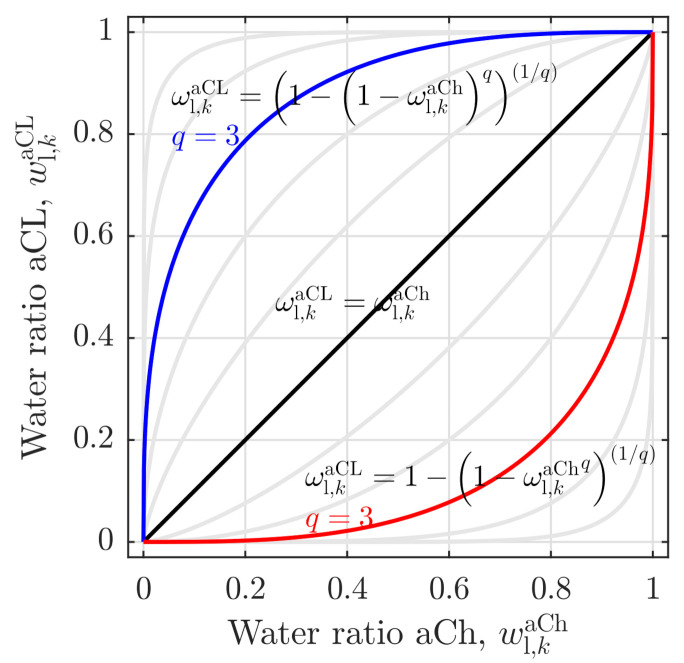
Coupling functions of the liquid phase ratio in the anode channel, $\omega_{l,k}^{\mathrm{aCh}}$ with the interfacial liquid phase volume ratio on the anode catalyst layer surface, $\omega_{l,k}^{\mathrm{aCL}}$.

Second, the coupling function between the channel and the CL water ratio is introduced. Here, Equation (40) shows a right-bent trend (s. Figure 3, blue line), for which the liquid phase ratio of the channel is always smaller or equal to the CL liquid phase ratio ($\omega_{l,k}^{\mathrm{aCh}}\leq \omega_{l,k}^{\mathrm{aCL}}$):

(40) $$\omega_{l,k}^{\mathrm{aCL}}={\left(1-{\left(1-\omega_{l,k}^{\mathrm{aCh}}\right)}^{q}\right)}^{\left(1/q\right)}$$

Dependent on the exponent *q*, the curve can either tend towards a linear coupling for q→1 (s. black line in Figure 3), or towards a step function for q→∞ (s. light gray lines above the black line in Figure 3). In addition, the left-bent inverse function (s. red line in Figure 3) is presented as a third coupling option. It is assumed that such unconventional coupling options lead to a reasonable correlation between the channel and the catalyst layer situations without an effusive model complexity, which will be validated in the following section.

All the model assumptions made here are based on the close integration of the model with the design of the experimental setup from our previous work [5]. The temperature control during the experiments justifies the assumptions of isothermal operation and, consequently, an isothermal model. Furthermore, the geometrical dimensions of the experimental setup (50 cm channel length, ≈200 μm sandwich thickness) allow for neglect along the channel transport in the MEA when identical transport parameters (diffusivity and proton conductivity) are assumed in sandwich and along the channel coordinate.

The resulting model, is validated with the experimental data in the following section and is used afterward for deeper analyses of the low stoichiometry operation mode.

## 3. Results and Discussion

### 3.1. Experimental Validation

The model is validated by two central results from the experiment: the polarization curve and the current density distribution.

#### Polarization Curve

**Figure 4:**
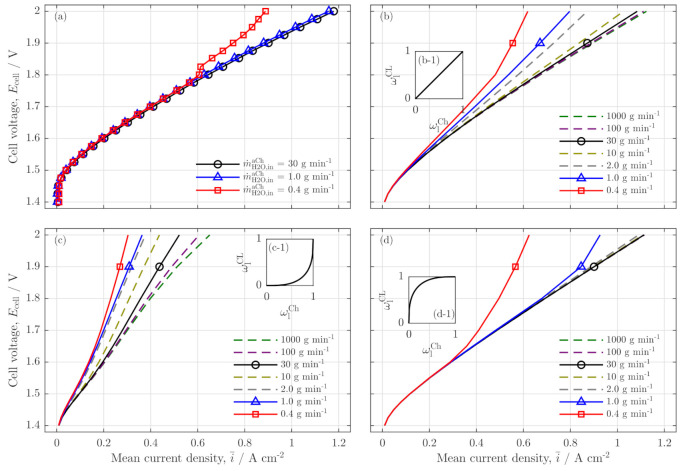
Polarization curves of (**a**) the experimental study (data from Immerz et al. [5], brief description of the experimental setup in Appendix C) with anode inlet water flow rates of $30\;g\min^{-1}$, $1.0\;g\min^{-1}$ and $0.4\;g\min^{-1}$ and (**b**–**d**) the model results for those flow rates as full lines with markers with different coupling functions (e.g., Equations (40)); (colored, dashed lines as additional flow rates). (**b**) linear coupling of liquid phase ratios (s. inset (b-1)), (**c**) left-bent coupling (s. inset (c-1)) and (**d**) a right-bent coupling (s. inset (d-1)) for q=3; temperature $T=60\;{}^{\circ }C$, ambient cell pressure $p^{\mathrm{aCh}}=p{}^{\mathrm{cCh}}=1\mathrm{bar}$.

First, in Figure 4,the polarization curves of the experiment and the model are compared. Figure 4a shows two polarization curves of water flow rates that are sufficiently high for a stable electrolysis operation (black circles and blue triangles) and one low water flow rate polarization curve (red squares) that reaches low stoichiometry operation at $\bar{i}\geq 0.6A{\mathrm{cm}}^{-2}$. In comparison, the other plots Figure 4b–d show simulation results for the three different coupling functions described in Figure 3. For the model, the same water flow rates as in the experiment were chosen (lines with circle, square, and triangle) supplemented by further flow rates to evaluate the experimental trends beyond the given data sets.

The validation aims to find the best qualitative accordance between the model results and the experiment. Therefore, the three coupling functions are compared, and the best fitting coupling is chosen for further analyses. The linear coupling in Figure 4b shows a qualitatively good accordance for the highest water flow rate ($30\;g\min^{-1}$, black circle). However, in comparison to the experiment, the polarization curve at $1.0\;g\min^{-1}$ is significantly different. While the experiment shows a quite identical polarization behavior for all flow rates above a critical stoichiometric water ratio, this coupling demonstrates a strong dependency from the water flow rates even for high stoichiometric operation.

A similar trend is given in the left-bent coupling function (s. Figure 4c and inset (c-1)). Additionally, the left-bent coupling increases the steep of the polarization curves and shows the quantitatively worst performance. Both, the linear and the left-bent coupling are considered insufficient to reflect the experimental data.

In contrast, the right-bent coupling (s. Figure 4d and inset (d-1)) adequately resembles the experimental measurements. First, for high water stoichiometry (s. black circle line) the polarization curve is identical with the experimental data and does not change much for a wide range of flow rates (s. dashed lines for $2.0\;g\min^{-1}\cdots 1000\;g\min^{-1}$). Furthermore, the low flow rate polarization curves ($1.0\;g\min^{-1}$, blue line; $0.4\;g\min^{-1}$, red line) show the experimentally observed strong and sudden deviation from the common trend, when a critical stoichiometry is reached.

However, the model data show that the drifting away of the polarization already occurs for the $1.0\;g\min^{-1}$ case (blue triangle). This indicates an imperfect parameter choice of the not parameterized model, leading to higher stoichiometry ratios, for which a critical limitation is reached. This is further observed in the $0.4\;g\min^{-1}$ case (red square), in which a critically low stoichiometry is reached at $\bar{i}=0.3\;A{\mathrm{cm}}^{-2}$, whereas, for an identical water flow rate, the experiment shows a drift away at $\bar{i}\approx 0.55\;A{\mathrm{cm}}^{-2}$ [5].

However, due to the good qualitative accordance between the model results in Figure 4d and the experimental data (s. Figure 4a), the following validation of the current density profiles was performed with the right-bent coupling function solely.

#### Validation of Current Density Distribution Profiles

**Figure 5:**
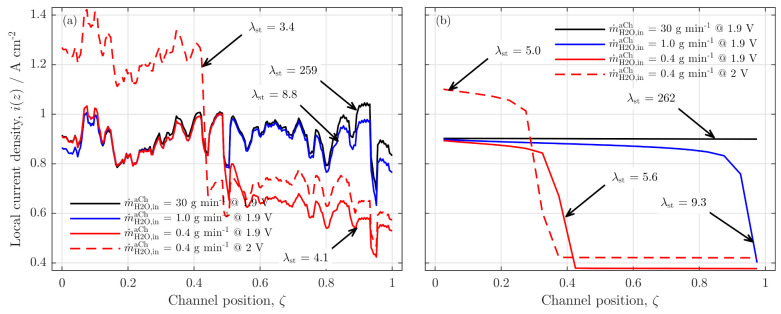
Current density distributions for different anode feed water flow rates; (**a**) results from the experimental study (data from Immerz et al. [5]), flow rates: $30\;g\min^{-1}$, black line; $1.0\;g\min^{-1}$ blue line, $0.4\;g\min^{-1}$, red lines (full at 1.9V, dashed at 2.0V); and (**b**) model results with a right-bent coupling between the liquid phase ratios in the channel and the CLs for identical flow rates and voltages; temperature, $T=60\;{}^{\circ }C$; ambient cell pressure, $p^{\mathrm{aCh}}=p^{\mathrm{cCh}}=1\mathrm{bar}$.

Figure 5a shows the experimentally observed current density profiles and Figure 5b the simulation results for the same cell voltages and flow rates. In the experimental analysis, the current density was measured at 252 measurement points along the 50cm long channel coordinate (experimental data from [5]). For the simulation, only 20 points along the *z*-axes were chosen and presented on the normalized channel position, ζ.

The idea of this figure is the quantitative comparison between the experimental and the model results. A comparison of both plots shows first of all the strong fluctuations of the experimental results. These measurement inaccuracies can clearly not be observed in the model results. Hence, only a qualitative trend is derived from the experimental results. For the high water flow rates ($30\;g\min^{-1}$ and $1.0\;g\min^{-1}$), the current density is, on average, quite homogeneous from the inlet region (ζ=0) to the outlet region (ζ=1) of the channel.

The model results show a similar trend for the highest flow rate (black line). With regard to the $1.0\;g\min^{-1}$ profile (blue line), a sharp decrease in the profile is visible at the channel outlet. A further reduction to $0.4\;g\min^{-1}$ (red line) amplifies this effect and shifts the current density drop to the middle region of the channel (s. red full line). The same effect was observed in the experimental data. While the current density is almost identical with the higher flow rate cases for the first half of the cell, the current density strongly decreases in the middle of the cell and stabilizes at a significantly lower level.

A quantitative difference between the model and experiment is the stoichiometric water ratio $\lambda_{\mathrm{st}}$ at which the current density profile begins to drop. While the experimental results show a homogeneous profile for $\lambda_{\mathrm{st}}=9$, the model data already reached a critical level and began to drop in the cell’s outlet region.

This quantitative difference is due to the fact that the model is not totally parameterized to the experiments. However, it is evident for the model and the experiment that the current density drops occur below a certain stoichiometric water ratio and that the position of this drop is shifted towards the inlet region with lower $\lambda_{\mathrm{st}}$.

Qualitatively, the experimental and the model data show a very good accordance. For high water flow rates, the current density is distributed homogeneously, and below a critical level, a sharp drop occurs beginning at the outlet region of the cell. Therefore, the validation is considered successfully, and further analyses are performed with the model.

### 3.2. Further Analysis of Low Stoichiometry Operation

**Figure 6:**
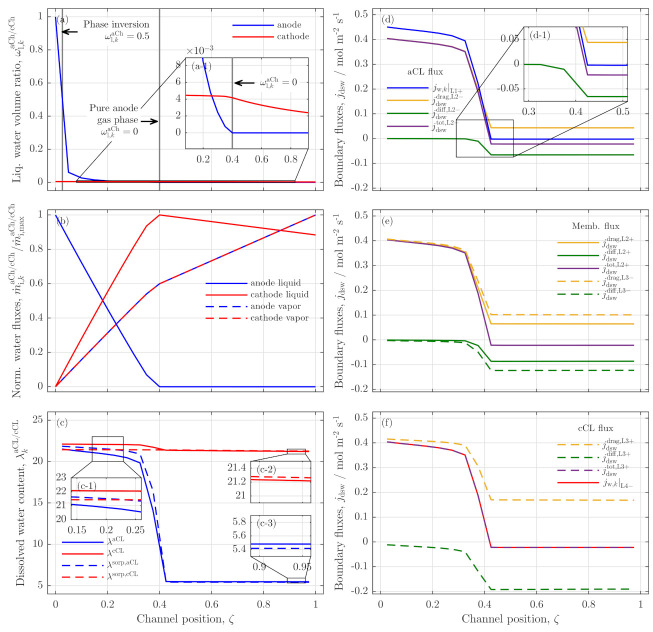
Along the channel distributions at conditions $E_{\mathrm{cell}}=1.9V$, ${\dot{m}}_{H}2O,{\mathrm{in}}^{aCh}=0.4\;g\min^{-1}$, $T=60\;{}^{\circ }C$, ambient pressure of (**a**) liquid water volume ratios, $\omega_{l,k}^{\mathrm{aCh}/\mathrm{cCh}}$; (**b**) normalized water flow rates of anode and cathode liquid water and vapor, ${\dot{m}}_{j,k}^{\mathrm{aCh}/\mathrm{cCh}}/{\dot{m}}_{j,\max}^{\mathrm{aCh}/\mathrm{cCh}}$ with j: l, vap; (**c**) dissolved water content in the catalyst layers, $\lambda_{k}^{\mathrm{aCL}/\mathrm{cCL}}$ and reference sorption water ratios (dashed lines, $\lambda^{\mathrm{sorp}}$); (**d**) water fluxes towards the aCL boundaries (liquid across L1, only dissolved water towards L2); (**e**) dissolved water fluxes towards the membrane boundaries L2 and L3; and (**f**) water fluxes towards cCL boundaries L3 for dissolved fluxes and liquid water across L4 in the cCL.

In the following, the model is used to analyze various distributed state variables at low stoichiometry conditions, which are not experimentally accessible. The analyses are performed with the data from the $0.4\;g\min^{-1}$ case at a cell voltage of 1.9V (s. Figure 5b, full red line), which are summarized in Figure 6. Figure 6a presents the liquid water volume ratio along the anode (blue line) and cathode channel (red line). The anode volume ratio drastically decreases from a full liquid flow at the entrance ($\omega_{l}^{\mathrm{aCh}}(\zeta =0)=1$) and shows a phase inversion from bubble flow to mist flow at a channel position of ζ≈0.025 (left gray vertical line).

Between positions 0.025–0.4 the liquid water ratio approaches zero and, as can be seen in inset (a-1), remains zero for the rest of the channel. In contrast, the cathode channel liquid volume ratio (red line) is constant up to ζ≈0.4 and starts to decrease when the water volume ratio at the anode is zero. The results indicate, that a phase inversion at position ζ=0.025 does not influence the current density distribution significantly, since the drastically reduced current density occurs at position ζ=0.4.

A visual analysis regarding the changes in two-phase flow pattern in the anode channel (experimentally tried in previous works [4,13]) can, therefore, not be used as reliable indication of drastic changes in the current density distribution. Consequently, a change of the two-phase flow regime cannot explain the current density drop in the middle region of the cell; however, the absence of liquid water in the anode channel can, when $\omega_{l}^{\mathrm{aCh}}\left(\zeta =0.4\right)=0$.

This absence of anode liquid water is also presented in Figure 6b, displaying all normalized gaseous and liquid water flow rates along the anode and cathode channels. The full blue line represents the liquid water flow rate in the anode channel, which decreases almost linearly from the anode inlet to ζ=0.4 as it supplies the feed water for the OER. Furthermore, a dissolved water flux migrates towards the cathode channel via drag and evaporates partly in the respective catalyst layers, leading to an almost linear increase of cathode liquid water and vapor flow rates in the channels up to position ζ=0.4.

Behind, the anode liquid feed is fully exploited, and the liquid water flow remains zero. However, due to a net water flow from the anode to the cathode up to position ζ=0.4, a reservoir of water has built up, which is now consumed from the cathode. As can be seen in the full red line, the cathode water has reached its maximum at ζ=0.4 and decreases nearly linearly towards the outlet region of the cell.

Although a feed from the vapor phase could be possible, the model results show a constant yet flatter increase from position ζ=0.4 on, caused by a lower current density and a lower production rate in the second half of the cell (s. dashed lines). The gases stay almost fully humidified (relative humidity ≥99) along the total channel length. The results indicate, that the OER in the outlet region is supplied from a cathode water reservoir, which was built up in the inlet region of the cell by a net water flow from the cathode towards the anode.

To support this hypothesis, Figure 6c shows the water ratios of both catalyst layers $\lambda^{\mathrm{aCL}/\mathrm{cCL}}$ along the channel, together with their theoretical sorption water ratios $\lambda^{\mathrm{sorp}}$ (dashed lines). If $\lambda <\lambda^{\mathrm{sorp}}$, than water is adsorbed into the particular catalyst layer, if $\lambda >\lambda^{\mathrm{sorp}}$, than water is desorbed. In the inlet region of the cell (s. inset (c-1)), the water is adsorbed by the anode side catalyst layer, while, on the cathode side, dissolved water is desorbed. When all the liquid anode water is depleted, the water ratio in the aCL drops rapidly, while only a little drop is seen in the cCL. The two insets (c-2) and (c-3) show that the sorption direction is reversed in this region: the cathode catalyst layer is now adsorbing water, while desorption occurs on the anode.

In Figure 6d–f, the resulting water fluxes (liquid and dissolved) are presented, resulting from the water ratio gradients. Here, Figure 6d shows the fluxes into and out of the aCL, Figure 6e shows the fluxes across the membrane boundaries, and Figure 6f shows the fluxes into/out of the cCL. Positive defined fluxes describe a flow from anode to cathode, negative fluxes describe the opposite direction. Looking at Figure 6d, the blue line represents the liquid water, which is fed from the anode channel to the reaction zone. While this flux is the highest flux by amount up to channel position ζ=0.4, it drops to slightly below zero behind that position (s. inset (d-1)).

A tiny dissolved water flux from the membrane to the aCL is observed, which fully desorbs there and leaves the aCL as a vapor phase across boundary L1 (s. Figure 2). In Figure 6d, the yellow line represents the drag driven flux, green is the diffusion driven flux, and the purple line is the sum of both, a total net water flux across the boundary L2.

Typically, the drag flux is dominating, leading to a net water flux from aCL to cCL. However, when the liquid water flux from the anode channel is vanished, and the concentration gradient (respectively the gradient of water ratios) from cathode to anode is high (s. Figure 6c), the back diffusion (green line) increases significantly by amount, and the water drag towards the cathode is drastically reduced. In total, the net flux is reversed and a diffusion dominated dissolved water flux feeds the OER by water transport from the cathode via the membrane into the aCL (s. Figure 6e,f)

The model results clearly indicate that the operation under low stoichiometry conditions leads to a reversal of the internal water fluxes, when the anode feed water is fully consumed. The reservoir that is built up to a net drag flux in the inlet region of the cell can serve as feed water in the outlet region and enable electrolysis reaction there but with strongly reduced performance.

### 3.3. Local Cell Potential Analysis

**Figure 7:**
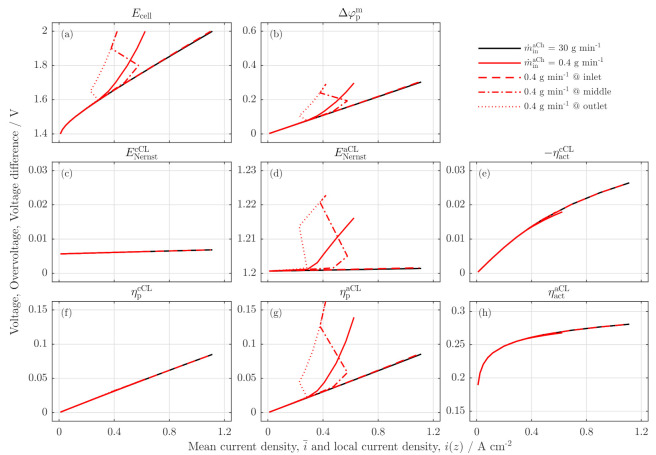
Cell voltage loss breakdown for reference scenario at $30\;g\min^{-1}$ (black full line) and a low stoichiometry operation at $0.4\;g\min^{-1}$ for full cell (full red lines) and locally: at the inlet (dashed red lines), the middle region of the cell (dash-dotted red line) and the outlet region of the cell (dotted red lines). (**a**) Cell voltage, (**b**) ohmic membrane voltage difference, (**c**) Nernst potential at the cathode, (**d**) Nernst potential at the anode, (**e**) cathode activation overpotential, (**f**) cathode proton potential, (**g**) anode proton potential and (**h**) anode activation overpotential.

The performance losses are analyzed in detail in Figure 7. Here, all voltages, potentials and overpotentials are investigated locally for the low stoichiometry operation and compared with a reference case of $30\;g\min^{-1}$ anode feed (black line). The full lines in Figure 7 show the overall potentials, the dashed line is the potential in the inlet region, the dash-dotted line is for the middle region, and the dotted line is the potential at the outlet region of the cell. For the cell voltage in Figure 7a, a low stoichiometry operation shows an overall drift away from the reference polarization curve for $\bar{i}\geq 0.3\;A{\mathrm{cm}}^{-2}$ (full red line).

Similar to the experimental data [5], the local profile at the inlet region shows a typical polarization curve that is identical to the reference case. For the middle and outlet region, an s-shaped dry-out behavior is observed, first in the outlet and for higher current densities in the middle region as well. Due to the reduced water ratio in the outlet region of the membrane, the Ohmic resistances of the membrane increase there first when low stoichiometry conditions are reached. This distinctive point propagates forward in the channel as the current density increases (s. Figure 7b).

The breakdown shows, furthermore, that the low stoichiometry operation has no or only a minor effect on all cathodic voltage losses, $E_{\mathrm{Nernst},k}^{\mathrm{cCL}}$, $\eta_{\mathrm{act},k}^{\mathrm{cCL}}$, $\eta_{p,k}^{\mathrm{cCL}}$ (s. Figure 7c,e,f) and the activation on the anode side, $\eta_{\mathrm{act},k}^{\mathrm{aCL}}$, s. Figure 7h). In literature, the dry-out behavior is explained by a gas accumulation in the aCL layer, leading to higher activation overpotentials [21].

However, in this model, the activation itself is independent from the anode fluid concentrations. Instead, the anode side Nernst potential (s. Figure 7d) depends on the water and gas concentration, which are distributed as highly inhomogeneous along the channel under low stoichiometry operations. Due to the low water concentration at the cell outlet region, the anode Nernst potential strongly increases. According to our experimental findings, the feed water transport into the reaction zone is integrated in the sorption kinetics, leading to an decrease of the anode proton conductivity (s. Figure 7g), which is typically observed as a mass transfer loss by electrochemical impedance spectroscopy [5].

The cell voltage breakdown supports the hypothesis form the experimental paper that a decreased water content in the membrane, and in the aCL are the main effects of the low stoichiometry operation. Additionally, compared to the experimental findings, the simulation results reveal an increased Nernst potential on the anode side, which contributes to the losses under low stoichiometry conditions, while the remaining overpotentials are independent.

### 3.4. Parameter Variation

Additionally, the model is used to evaluate the effect of operational parameters and modes that cannot be achieved in the experiment during the low stoichiometry operation. Furthermore, effects on the safe operation of a PEMWE cell are concerned under low stoichiometry.

#### Influences of Pressure, Temperature and Membrane Thickness

**Figure 8:**
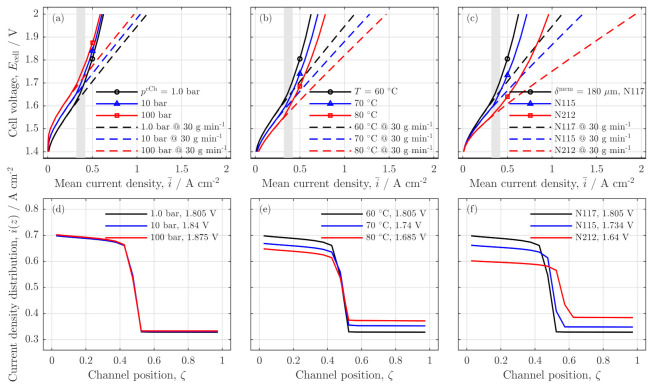
Polarization curves for different operating parameters: (**a**) cathode pressure, (**b**) cell temperature and (**c**) the membrane thickness for a sufficient water flow rate of $30\;g\min^{-1}$ (dashed lines) and a low water inlet flow rate of $0.4\;g\min^{-1}$ (full lines). The gray vertical strip indicates the current density range at which the cell starts to dry out. Current density distributions at $0.4\;g\min^{-1}$ and a mean current density of $\bar{i}=0.5\;A{\mathrm{cm}}^{-2}$ for a variation of (**d**) cathode pressure, (**e**) cell temperature and (**f**) membrane thickness.

First, in Figure 8, the influences of cathode pressure (Figure 8a), cell temperature (Figure 8b) and membrane thickness (Figure 8c) are investigated for the low stoichiometry operation with an anode feed of $0.4\;g\min^{-1}$. Polarization curves for a sufficiently high flow rate of $30\;g\min^{-1}$ (dashed lines) are used as reference. Regarding those, the expected trends were observed: for higher cathode pressures, the polarization curve is slightly raised because of an increased cathode side Nernst potential [22] (s. Figure 8a).

Positive effects on the cell performance induced by higher pressure and reducing this voltage raise, could also be already observed but are not reflected in the model formulation used here. Furthermore, a flatter polarization curve is achieved on the one hand with higher temperatures (s. Figure 8b) [23] primarily due to improved proton conductivity of the membrane and an improved activation of the reactions and on the other hand by a reduced membrane thickness due to a reduced proton resistance of a thinner membrane [24,25] (s. Figure 8c).

Figure 8 shows that the all parameter variations led to a similar trend under low stoichiometry operation (full lines). As the gray vertical stripes indicate, the polarization curves drift away from the reference when water stoichiometry reaches a level of $\lambda_{\mathrm{st}}=7.5$–10. For all parameter variations, the current density distribution under starvation conditions (${\dot{m}}_{H2O}^{\mathrm{aCh}}=0.4\;g\min^{-1}$, $\bar{i}=0.5\;A{\mathrm{cm}}^{-2}$) is presented in Figure 8d–f.

While the polarization curves show that the appearance of the dehumidification is achieved with all tested parameters, the current density distribution at an identical mean current density reveals differences in the distributions. The cathode pressure variation (Figure 8d) shows that all distributions are identical while only the cell voltage is increased with higher pressures due to an increased cathode Nernst potential. In Figure 8e, the temperature increase leads to a reduction in the current density step size at the position of anode channel water absence.

This can be explained with the improved proton conductivity at higher temperatures (s. Equation (16)) and the more homogeneously distributed dissolved water concentration in all CCM layers ($c_{\mathrm{dsw},k}^{\mathrm{aCL}/m/\mathrm{cCL}}$). When the dissolved water concentration of the anode catalyst layer is higher, the conductivity increases coherently with an increase of the local current density. Consequently, to achieve the identical mean current density between the higher and lower T case, the current density in the inlet region can be lower.

For thinner membranes (s. Figure 8f), the reduced resistance effect is present in combination with an improved back diffusion flux due to the reduced membrane thickness. This combination shows a less pronounced step in the current density distribution as well as a shift of the step towards the outlet region of the cell because liquid water is available in the anode channel up to a slightly further back channel position.

#### Influence on Safety and Crossover

In the following, safety issues of the low stoichiometry operation are studied with regard to the gas crossover. Typically, crossover is an issue for high differential pressures and thin membranes [26,27]. Hence, cases for the thin Nafion^®^ N212 membranes and cathode pressures of 50 bar and 100 bar are compared with the low stoichiometry operation at reference conditions (ambient pressure and Nafion^®^ N117).

**Figure 9:**
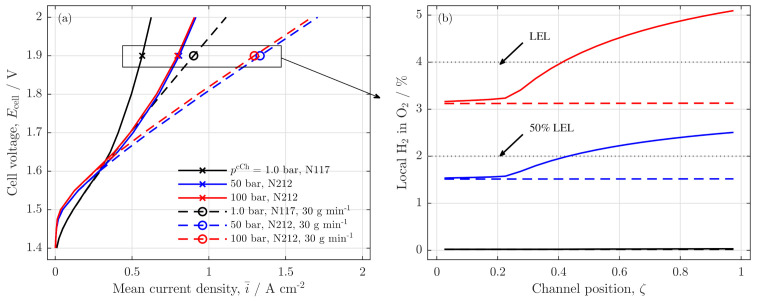
Safety and crossover analysis: (**a**) polarization curves at ambient ($p^{\mathrm{cCh}}=1.0\;\mathrm{bar}$) and at increased cathode pressures (50 bar and 100 bar) with different membrane thicknesses (N117: $\delta_{m}=180\;\mu m$, N212: $\delta_{m}=50.8\;\mu m$) for water feeds of $30g\min^{-1}$ (dashed lines with circles) and low water feeds of $0.4\;g\min^{-1}$ (full lines with crosses); $T=60\;{}^{\circ }C$. (**b**) Local volume ratio of H_2_ in O_2_ along the anode channel for the different setups at a common cell voltage, $E_{\mathrm{cell}}=1.9\;V$.

First, Figure 9a shows the expected polarization curves for thinner membranes and high cathode pressures with high water fluxes (dashed lines): the polarization behavior and the system performance is improved in comparison to the reference case for $\bar{i}\geq 0.3\;A{\mathrm{cm}}^{-2}$. A similar trend is observed for the low water feed cases. However, for all low water feed cases, the starvation effect is present as expected.

To analyze the safety issue of the starvation case, the local hydrogen in oxygen volume ratio along the channel coordinate is plotted for all presented cases at cell voltage, $E_{\mathrm{cell}}=1.9V$ (s. Figure 9b). The high water feed cases (dashed lines) show a constant trend along the channel. However, as expected, the elevated cathode pressure and the reduced membrane thickness increase the hydrogen content in the gas phase of the anode channel, which can exceed 50% of the lower explosion limit (LEL) in the case of $p^{\mathrm{cCh}}=100\;\mathrm{bar}$ (dashed red line).

When the water feed is additionally reduced to a low water stoichiometry operation, a strong increase in the H_2_ in O_2_ ratio is observed beginning at the channel position, where the anode water feed is exhausted (full blue and red line). For the 50 bar case, this leads to local H_2_ in O_2_ ratios above the 50% LEL along the outlet half of the cell, while the 100 bar case even exceeds the full LEL in the outlet region of the cell. Particularly with regard to the absence of liquid water, this gas composition can clearly pose a safety issue.

### 3.5. Remarks on Low Stoichiometry Operation

Finally, this analysis also provides a critical evaluation of the low stoichiometry operation. The model assumptions made are based on previous experimental data [5] with the central assumption that the cell operates fully isothermally. This is reasonable for the experimental setup used in [5], because the integrated heating/cooling system allows a good temperature management.

**Figure 10:**
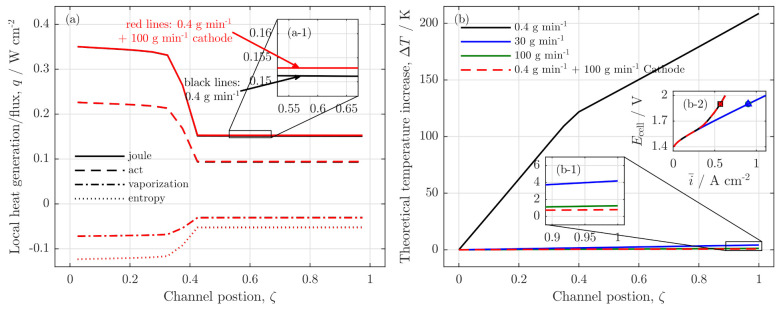
Thermal analysis of the low stoichiometry operation at $E_{\mathrm{cell}}=1.9\;V$ and ${\dot{m}}_{H_{2}O,\mathrm{in}}^{aCh}=0.4\;g\min^{-1}$; (**a**) heat sources along the channel for cases with and without cathode cooling water flow; (**b**) theoretical temperature increase in the cell along the channel for different flow rates, (b-2) polarization curves calculated isothermally for the different cases; marker on the polarization curve indicate the operation point for the local analyses.

Consequently, this assumption was applied to the model. To assess this assumption for real applications, a simple thermal approximation is performed (s. Equations (A40)–(A47)). The calculations include Joule heat generation, heat generation by the activation, heat demand by entropy change and the latent heat demand of the vaporization. In Figure 10a, the local sources and sinks are presented along the channel. Since all of those processes are directly or indirectly connected with the current density distribution, the source/sink profiles show the typical drop at channel position ζ=0.4 as well.

The resulting theoretical temperature increase is plotted in Figure 10b, with the assumption that the produced heat is only removed by convective transport of the channel fluids, which should not be the case for real applications [28]. As can be seen in the full black curve, the low water feed case would theoretically exceed a temperature increase of 200K; thus, the experimental analysis would not have any practical relevance.

In contrast, the high water flow rates (blue and green lines) show only a minor temperature increase of 4K (b-1) in Figure 10b, which is in a technically relevant range but does not show any starvation behavior (s. green and blue polarization curves in (b-2) of Figure 10). However, the starvation observation can have technical relevance when the cell or stack cooling is realized otherwise, as the dashed red line indicates. Here, the anode water feed is low (${\dot{m}}_{H}2O,{\mathrm{in}}^{aCh}=0.4\;g\min^{-1}$), and a high water flow rate is set on the cathode (${\dot{m}}_{H}2O,{\mathrm{in}}^{cCh}=100\;g\min^{-1}$).

The inset (b-1) shows tolerable temperature increase of 1 K, while the polarization behavior is identical as in the only anode fed case (b-2) in Figure 10, which shows a water shortage in the anode outlet region. With regard to the heat generation, the simple model reveals only slight differences in the pure anode feed case and in the anode feed and cathode cooled case (a-1) in Figure 10.

Generally, an operation of PEMWE cells or systems is performed with high water flow rates and a high water stoichiometry in order to control mainly the thermal cell behavior [29]. However, the experimental findings indicate that the pure electrochemical behavior of the cell is only slightly influenced as long as there is liquid water available in the anode channel. When there is a water shortage in the outlet region of the cell, but a homogeneous temperature distribution can be achieved by cathode side cooling for example, the model shows that a low water stoichiometry can become a technical issue that needs to be avoided.

## 4. Conclusions

In the present work, an isothermal steady state 1+1-d proton exchange membrane water electrolysis (PEMWE) model was discussed in order to investigate local phenomena in a PEMWE cell under low stoichiometry conditions in the context of our previous experimental work [5]. The model consists of a sub-model in sandwich direction based on Trinke [15] and an along the channel sub-model, capable of illustrating the local distribution of various state variables and fluxes.

The validation with the polarization curve and the current density distribution proved that the model was able to describe the experimental findings for high and low water stoichiometry. Furthermore, the model results show that the oxygen evolution reaction in the outlet region of the anode channel can be fed by water from the cathode side when the anode channel is dry. The water feed by vapor shows minor relevance. The local polarization analysis indicates that the low stoichiometry mode reduces the proton conductivity in the membrane and anode catalyst layer and increases the anode Nernst potential.

The presented analysis serves as a description of the experimental findings and enlightens potential water transport and operation methods in an unconventional PEMWE operation mode. Although low stoichiometry operation is not a favorable mode in the conventional PEMWE setup, these analyses help to improve the understanding of water transport inside the cell. Further they prove how special conditions can be set, which may be required for analyses dedicated to degradation aspects, i.e., dry out scenarios.

With regard to technologies other than liquid feed PEMWE, the findings may be of interest for vapor feed operation or system setups with bipolar membranes in which water management is of high importance.

## Figures and Tables

**Table 1:** Overview on the model equation system.

Charge balances	
Electron potential anode, state equations	Equations (1)–(3)
Proton potential anode, state equations	Equations (4)–(6)
Proton potential membrane, state equations	Equations (7)–(9)
Constitutive and closing equations	Equations (10)–(16)
Electron potential cathode, state equations	Equations (A20)–(A22)
Proton potential cathode, state equations	Equations (A23)–(A25)
Additional electrical equations	Equations (A1)+(A2)
**Dissolved gases**	
H_2_, O_2_ concentrations anode, state equations	Equations (17)–(19)
H_2_, O_2_ concentrations membrane, state equations	Equations (20)–(22)
Constitutive and closing equations	Equations (23)–(26)
H_2_, O_2_ concentrations cathode, state equations	Equations (A26)–(A28)
Additional dissolved gases equations	Equations (A3)–(A7)
**Dissolved water**	
Water concentrations anode, state equations	Equations (27)–(29)
Constitutive and closing equations	Equations (30)–(33)
Water concentrations membrane, state equations	Equations (A29)–(A31)
Water concentrations cathode, state equations	Equations (A32)–(A34)
Additional dissolved water equations	Equations (A8)–(A15)
**Channel model**	
Channel fluxes, state equations	Equations (34)–(36)
Constitutive and closing equations	Equations (37)–(38)
Coupling equations	Equations (39)–(40)
Channel volume fluxes	Equations (A16)–(A19)

## Data Availability

Not applicable.

## Appendix A. Model

The model is set up in the sandwich direction as system of 9 potential state functions; 6 charge flux state functions; 15 state functions for concentrations of the dissolved substances hydrogen, oxygen and water; and 12 state functions for the dissolved flux densities. Furthermore, four state functions for liquid water, hydrogen, oxygen and vapor are applied for both the anode and cathode channel in each element. The model has six adjustable variables (namely: the electron potential on the anode side, inlet water flux, temperature, anode and cathode pressure and thickness of the membrane). All fixed parameters are shown in the following section and in Table A3.

In the following sections, the remaining equations of the balance model in the sandwich direction (s. Table A1) and the channel coordinate (s. Table A2) are presented. Furthermore, necessary model equations, independent form the state variables are described. Finally, the calculations for a simple thermal approximation model are given.

The sandwich model is set up for the protons and electrons. Furthermore, dissolved gases (hydrogen, oxygen and vapor) and dissolved water is modeled in the sandwich direction. Along the channel coordinate in the anode and cathode channel, water is balanced in liquid and gaseous form. Furthermore, the gases hydrogen and oxygen are balanced.

Sandwich Model Equations

In Table A1 the remaining sandwich model equations for the cathode catalyst layer and membrane are given, which are analogous to the model equations in Section 2.1.

Channel Model Equations

Table A2 presents the remaining determination equations for the channel model for the cathode channel (s. Section 2.2).

Electrical Model

The electrical model is extended by a temperature dependency of the Nernst equation based on the NIST data [30]. The temperature correction is per definition only applied for the oxygen evolution reaction.

(A1) $$E_{0}^{\mathrm{aCL}}=1.478\;V-8.347\;\cdot \;T\frac{V}{K}$$

Additionally, the exchange current densities for both half cell reactions $i_{0}^{v}$ are calculated by a temperature dependency as follows

(A2) $$i_{0}^{v}=i_{0,\mathrm{ref}}^{v}\;\cdot \;\exp\left[\frac{E_{A}^{v}}{R}\;\cdot \;\left(\frac{1}{300}-\frac{1}{T/K}\right)\right]\;v:\mathrm{aCL},\mathrm{cCL}$$

where $i_{0,\mathrm{ref}}^{v}$ is the reference exchange current density and $E_{A}^{v}$ is the activation energy.

Dissolved Gases Model

In the dissolved gases model, the transport is pure diffusion, for which the effective dissolved gas diffusity, $D_{\mathrm{dsg},\mathrm{eff},j}^{v}$, is described as

(A3) $$D_{\mathrm{dsg},\mathrm{eff},j}^{v}=0.42\frac{\epsilon_{\mathrm{ion}}^{v}}{\tau_{\mathrm{ion}}^{v}}\;\cdot \;D_{\mathrm{dsg},j,0}\;\cdot \;\exp\left(\frac{E_{A,j,0}}{RT}\right)\;v:\mathrm{aCL},\mathrm{cCL}\;j:O_{2},H_{2}$$

Here, $D_{\mathrm{dsg},j,0}$ is a standard diffusity, which is temperature corrected. The temperature correction is realized, with an Arrhenius approach and the Activation energy $E_{j,0}$. The saturation concentration for hydrogen in the cathode, $c_{\mathrm{dsg},H2}^{\mathrm{cCL},\mathrm{sat}}$ and oxygen in the anode, $c_{\mathrm{dsg},O2}^{\mathrm{aCL},\mathrm{sat}}$ is calculated as follows with Henry’s law.

(A4) $$\begin{array}{cc} c_{\mathrm{dsg},O2}^{\mathrm{aCL},\mathrm{sat}} & =\left(p^{\mathrm{aCL}}-p_{\mathrm{vap}}^{\mathrm{sat}}\right)\;\cdot \;S_{O2} \end{array}$$

(A5) $$\begin{array}{cc} c_{\mathrm{dsg},H2}^{\mathrm{cCL},\mathrm{sat}} & =\left(p^{\mathrm{cCL}}-p_{\mathrm{vap}}^{\mathrm{sat}}\right)\;\cdot \;S_{H2} \end{array}$$

Here, $p^{v}$ are the CL pressures, and $S_{j}$ are the solubilities for hydrogen and oxygen, with

(A6) $$\begin{array}{cc} S_{O2} & =0.55\;\cdot \;\exp\left[-66.73538+\frac{8745.547}{T/K}+24.45264\;\cdot \;\log\left(\frac{T/K}{100}\right)\right]\;\cdot \;\mathrm{mol}m^{-3}{\mathrm{Pa}}^{-1} \end{array}$$

(A7) $$\begin{array}{cc} S_{H2} & =0.55\;\cdot \;\exp\left[-48.1611+\frac{5528.45}{T/K}+16.8893\;\cdot \;\log\left(\frac{T/K}{100}\right)\right]\;\cdot \;\mathrm{mol}m^{-3}{\mathrm{Pa}}^{-1} \end{array}$$

based on the empirical equation of Ito et al. [31] and the formulation of Trinke [15]. The saturation concentrations for oxygen on the cathode, and hydrogen on the anode are zero due to the reference of a cell without crossover ($c_{\mathrm{dsg},O2}^{\mathrm{cCL},\mathrm{sat}}=c_{\mathrm{dsg},H2}^{\mathrm{aCL},\mathrm{sat}}=0$).

Dissolved Water Model

In the dissolved water model, a diffusive flux is used. The dissolved water diffusivity, $D_{\mathrm{dsw},\mathrm{eff}}^{v}$ is calculated with the an empirical equation from Trinke [15] fitted to the data of Zhao et al. [32]

(A8) $$D_{\mathrm{dsw},\mathrm{eff}}^{v}=\epsilon_{\mathrm{ion}}^{v}\;\cdot \;\left(3.6\;\cdot \;\lambda_{\mathrm{dsw}}^{\mathrm{sat}}-7.8\right)\times 10^{-6}\;\cdot \;\exp\left(\frac{3350}{T/K}\right)$$

Furthermore, the dependency between the water ratio, $\lambda_{k}^{v}$ and the dissolved water concentration is described here.

(A9) $$\lambda_{k}^{v}=c_{\mathrm{dsw},k}^{v}\;\cdot \;\frac{EW}{\rho_{\mathrm{dry}}}\;\cdot \;{\left(1-c_{\mathrm{dsw},k}^{v}\;\cdot \;\frac{{\tilde{M}}_{w}}{\rho_{l}}\right)}^{-1}\;v:\mathrm{aCL},m,\mathrm{cCL}$$

In Equations (A9), EW represents the equivalent weight of a Nafion^®^ membrane ($EW=1.1\mathrm{kg}\;{\mathrm{mol}}^{-1}$), $\rho_{\mathrm{dry}}$ is the dry density of the Nafion^®^ membrane ($\rho_{\mathrm{dry}}=2000\mathrm{kg}\;m^{-3}$), and $\rho_{l}$ is the density of liquid water $\rho_{l}=1000\mathrm{kg}\;m^{-3}$ [33]. The water ratios in the membrane and cathode catalyst layer are calculated analogously.

Vaporization

To calculate the adsorbed or desorbed vapor flux, the activity of vapor in the CLs $a_{\mathrm{vap},k}^{v}$ as function of the local vapor pressure $p_{\mathrm{vap},k}^{v}$ and the saturation pressure $p_{\mathrm{vap}}^{\mathrm{sat}}$ is used. It is assumed that the vapor pressure is identical in CL and channel.

(A10) $$a_{\mathrm{vap},k}^{v}=\frac{p_{\mathrm{vap},k}^{v}}{p_{\mathrm{vap}}^{\mathrm{sat}}}\;v:\mathrm{aCL},\mathrm{cCL}$$

The saturation pressure $p_{\mathrm{vap}}^{\mathrm{sat}}$ is calculated by the empirical Antoine equation [34],

(A11) $$\log\left(\frac{p_{\mathrm{vap}}^{\mathrm{sat}}}{10\times 10^{5}\mathrm{Pa}}\right)=4.6542-\frac{1435.264K}{T-64.848K}$$

The vapor pressure is calculated with Raoult’s law:

(A12) $$p_{\mathrm{vap},k}^{v}=\frac{v_{\mathrm{vap},k}^{v}}{v_{\mathrm{vap},k}^{v}+v_{O2,}^{v}+v_{H2,k}^{v}}\;\cdot \;p^{v}$$

The saturation vapor contents $\lambda_{\mathrm{vap},k}^{v}$ is calculated empirically (e.g., Springer et al. [17]).

(A13) $$\lambda_{\mathrm{vap},k}^{v}=\left(0.043+17.81a_{\mathrm{vap},k}^{v}-39.85{a_{\mathrm{vap},k}^{v}}^{2}+36.0{a_{\mathrm{vap},k}^{v}}^{3}\right)\;v:\mathrm{aCL},\mathrm{cCL}$$

The corresponding vapor saturation concentration, $c_{\mathrm{dsg},\mathrm{vap},k}^{v}$ (s. Equations (31) and (32)) is calculated similar to Equation (A9) as:

(A14) $$c_{\mathrm{dsg},\mathrm{vap},k}^{v}=\lambda_{\mathrm{vap},k}^{v}\;\cdot \;{\left(\frac{EW}{\rho_{\mathrm{dry}}}+\lambda_{\mathrm{vap},k}^{v}\;\cdot \;\frac{{\tilde{M}}_{w}}{\rho_{l}}\right)}^{-1}\;v:\mathrm{aCL},\mathrm{cCL}$$

Furthermore, the vaporization mass transfer coefficient $k^{\mathrm{vap}}$ is defined as:

(A15) $$k^{\mathrm{vap}}=\frac{Sh_{\mathrm{ec}}\;\cdot \;D_{\mathrm{vap}}}{d_{\mathrm{pore}}}$$

with $Sh_{\mathrm{ec}}$ is the dimensionless Sherwood number, $D_{\mathrm{vap}}$ the diffusivity of vapor, and $d_{\mathrm{pore}}$ the pore diameter of the CL (s. Table A3).

Channel Model

The calculation of the phase ratios $\omega_{l,k}^{\mathrm{aCh}}$ (s. Equation (39)) is dependent from the local volume flux densities, $v_{l,k}^{\mathrm{aCh}}$:

(A16) $$\begin{array}{cc} v_{l,k}^{\mathrm{aCh}} & =g_{l,k}^{\mathrm{aCh}}\;\cdot \;\rho_{l}/{\tilde{M}}_{l} \end{array}$$

(A17) $$\begin{array}{cc} v_{O_{2},k}^{\mathrm{aCh}} & =g_{O_{2},k}^{\mathrm{aCh}}\;\cdot \;RT/p^{\mathrm{aCh}} \end{array}$$

(A18) $$\begin{array}{cc} v_{H_{2},k}^{\mathrm{aCh}} & =g_{H_{2},k}^{\mathrm{aCh}}\;\cdot \;RT/p^{\mathrm{aCh}} \end{array}$$

(A19) $$\begin{array}{cc} v_{\mathrm{vap},k}^{\mathrm{aCh}} & =g_{\mathrm{vap},k}^{\mathrm{aCh}}\;\cdot \;RT/p^{\mathrm{aCh}} \end{array}$$

It is assumed that the fluids behave as ideal gases respectively as ideal liquid. Furthermore, it is assumed that the flux velocities are identical for all components.

**Table A1:** Additional balance equations for the sandwich model.

Unit	Equation
*Potential field electron conductor*	
$\varphi_{e,k}^{\mathrm{cCL}}$	(A20) $$0=-\left(i_{e,k}{|}_{L4^{-}}-0\right)+\delta^{\mathrm{cCL}}\sigma_{e,k}^{\mathrm{cCL}}$$
$i_{e,k}{|}_{L4^{-}}$	(A21) $$i_{e,k}{|}_{L4^{+}}=\kappa_{e}^{\mathrm{cCL}}\frac{\varphi_{e,k}{|}_{L4}-\varphi_{e,k}^{\mathrm{cCL}}}{\delta^{\mathrm{cCL}}/2}$$
$\varphi_{e,k}{|}_{L4}$	(A22) $$0=\varphi_{e}^{c},\mathrm{set}-\varphi_{e,k}{|}_{L4}$$
*Potential field proton conductor*	
$\varphi_{p,k}^{\mathrm{cCL}}$	(A23) $$0=-\left(0-i_{p,k}{|}_{L3^{+}}\right)+\delta^{\mathrm{cCL}}\sigma_{p,k}^{\mathrm{cCL}}$$
$i_{p,k}{|}_{L3^{+}}$	(A24) $$i_{p,k}{|}_{L3^{+}}=-\kappa_{p,\mathrm{eff},k}^{\mathrm{cCL}}\frac{\varphi_{p,k}^{\mathrm{cCL}}-\varphi_{p,k}{|}_{L3}}{\delta^{\mathrm{cCL}}/2}$$
$\varphi_{p,k}{|}_{L3}$	(A25) $$0=i_{p,k}{|}_{L3^{+}}-i_{p,k}{|}_{L3^{-}}$$
*Dissolved gases*	
$c_{\mathrm{dsg},j,k}^{\mathrm{cCL}}$	(A26) $$0=-\left(0-j_{\mathrm{dsg},j,k}{|}_{L3^{+}}\right)+\delta^{\mathrm{cCL}}\sigma_{\mathrm{dsg},j,k}^{\mathrm{evo}},\mathrm{cCL}-j_{jk}{|}_{L4^{-}}$$
$j_{\mathrm{dsg},j,k}{|}_{L3^{+}}$	(A27) $$j_{\mathrm{dsg},j,k}{|}_{L3^{+}}=-D_{\mathrm{dsg},\mathrm{eff},j}^{\mathrm{cCL}}\frac{c_{\mathrm{dsg},j,k}^{\mathrm{cCL}}-c_{\mathrm{dsg},j,k}{|}_{L3}}{\delta^{\mathrm{cCL}}/2}$$
$c_{\mathrm{dsg},j,k}{|}_{L3}$	(A28) $$0=j_{\mathrm{dsg},j,k}{|}_{L3^{-}}-j_{\mathrm{dsg},j,k}{|}_{L3^{+}}$$
*Dissolved water*	
$c_{\mathrm{dsw},k}^{m}$	(A29) $$0=-\frac{d}{dx}\left(-D_{\mathrm{dsw}},{\mathrm{eff}}^{m}\frac{dc_{\mathrm{dsw},k}^{m}}{dx}\right)-\frac{d}{dx}\left(-\frac{n_{\mathrm{drag},\mathrm{eff},i,k}^{m}}{F}\kappa_{p,\mathrm{eff},i,k}^{m}\frac{d\varphi_{p,k}^{m}}{dx}\right)$$
$j_{\mathrm{dsw},k}{|}_{L2^{+}}$	(A30) $$j_{\mathrm{dsw}}{|}_{L2^{+}}=-D_{\mathrm{dsw}},{\mathrm{eff}}^{m}\frac{c_{\mathrm{dsw},1,k}^{m}-c_{\mathrm{dsw},k}{|}_{L2}}{\Delta x^{m}/2}-\frac{n_{\mathrm{drag},\mathrm{eff},1,k}^{m}}{F}\kappa_{p,\mathrm{eff},1,k}^{m}\frac{\varphi_{p,1,k}^{m}-\varphi_{p,k}{|}_{L2}}{\Delta x^{m}/2}$$
$j_{\mathrm{dsw},k}{|}_{L3^{-}}$	(A31) $$j_{\mathrm{dsw},k}{|}_{L3^{-}}=-D_{\mathrm{dsw}},{\mathrm{eff}}^{m}\frac{c_{\mathrm{dsw},k}{|}_{L3}-c_{\mathrm{dsw},n,k}^{m}}{\Delta x^{m}/2}-\frac{n_{\mathrm{drag},\mathrm{eff},n,k}^{m}}{F}\kappa_{p,\mathrm{eff},n,k}^{m}\frac{\varphi_{p,k}{|}_{L3}-\varphi_{p,n,k}^{m}}{\Delta x^{m}/2}$$
$c_{\mathrm{dsw},k}^{\mathrm{cCL}}$	(A32) $$0=-\left(0-j_{\mathrm{dsw},k}{|}_{L3^{+}}\right)-j_{w,k}{|}_{L4^{-}}$$
$j_{\mathrm{dsw},k}{|}_{L3^{+}}$	(A33) $$j_{\mathrm{dsw},k}{|}_{L3^{+}}=-D_{\mathrm{dsw}},{\mathrm{eff}}^{\mathrm{cCL}}\frac{c_{\mathrm{dsw},k}^{\mathrm{cCL}}-c_{\mathrm{dsw},k}{|}_{L3}}{\delta^{\mathrm{cCL}}/2}-\frac{n_{\mathrm{drag},\mathrm{eff},k}^{\mathrm{cCL}}}{F}\kappa_{p,\mathrm{eff},k}^{\mathrm{cCL}}\frac{\varphi_{\mathrm{dsw},k}^{\mathrm{cCL}}-\varphi_{\mathrm{dsw},k}{|}_{L3}}{\delta^{\mathrm{cCL}}/2}$$
$c_{\mathrm{dsw},k}{|}_{L3}$	(A34) $$0=j_{\mathrm{dsw},k}{|}_{L3^{ȡ}}-j_{\mathrm{dsw},k}{|}_{L3^{+}}$$

**Table A2:** Additional cathode channel equations.

Unit	Equation
*Cathode channel fluxes*	
$g_{l,k}^{\mathrm{cCh}}$	(A35) $$0=g_{l,k-1}^{\mathrm{aCh}}-g_{l,k}^{\mathrm{cCh}}+\frac{{\left.j_{w,k}\right|}_{L}4+}{\delta^{\mathrm{cCh}}}\;\cdot \;\Delta z-\frac{j_{\mathrm{vap},k}^{\mathrm{cCh}}}{\delta^{\mathrm{cCh}}}\;\cdot \;\Delta z$$
$g_{j,k}^{\mathrm{cCh}}$	(A36) $$0=g_{j,k-1}^{\mathrm{cCh}}-g_{j,k}^{\mathrm{cCh}}+\frac{{\left.j_{j,k}\right|}_{L}4+}{\delta^{\mathrm{cCh}}}\;\cdot \;\Delta z$$
$g_{\mathrm{vap},k}^{\mathrm{cCh}}$	(A37) $$0=g_{\mathrm{vap},k-1}^{\mathrm{cCh}}-g_{\mathrm{vap},k}^{\mathrm{cCh}}+\frac{j_{\mathrm{vap},k}^{\mathrm{cCh}}}{\delta^{\mathrm{cCh}}}\;\cdot \;\Delta z$$
*Further equations*	
$j_{\mathrm{vap},k}^{\mathrm{cCL}}$	(A38) $$j_{\mathrm{vap},k}^{\mathrm{cCL}}=\delta^{\mathrm{cCL}}\;\cdot \;\frac{k^{\mathrm{vap}}a_{\mathrm{pore}}^{\mathrm{cCL}}}{RT}\left(p_{\mathrm{vap}}^{\mathrm{sat}}-p_{\mathrm{vap},k}^{\mathrm{cCh}}\right)$$
$\omega_{l,k}^{\mathrm{cCh}}$	(A39) $$\omega_{l,k}^{\mathrm{cCh}}=\frac{v_{l,k}^{\mathrm{cCh}}}{v_{l,k}^{\mathrm{cCh}}+v_{O_{2},k}^{\mathrm{cCh}}+v_{H_{2},k}^{\mathrm{cCh}}+v_{\mathrm{vap},k}^{\mathrm{cCh}}}$$

**Table A3:** Chosen parameters and constants.

Variable	Symbol	aCL	m	cCL	Unit	Source
Temperature	*T*		60		${}^{\circ }C$	
Pressure	*p*	1.0		1.0	bar	
Faraday’s constant	F		96,485		$A\;s\;{\mathrm{mol}}^{-1}$	
Layer thickness	$\delta^{v}$	5.432	180	5.556	μm	calc. in [15]
Electron conductivity	$\kappa_{e}^{v}$	22.2	-	25	$S\;m^{-1}$	[15]
Ex. current dens.	$i_{0,\mathrm{ref}}^{v}$	$3\times 10^{-4}$	-	700	$A\;{\mathrm{cm}}^{-2}$	chosen s. [35]
Ref. hydrogen conc.	$c_{\mathrm{dsg},H2}^{0}$			0.3921	$\mathrm{mol}/m^{3}$	calc. in [15]
Ref. oxygen conc.	$c_{\mathrm{dsg},O2}^{0}$	0.6368			$\mathrm{mol}/m^{3}$	calc. in [15]
Ref. liq. water content	$\lambda^{\mathrm{sat},l}$		22		$\mathrm{mol}/m^{3}$	[17]
Activation energy	$E_{A}^{v}$	54		54	$kJ\;{\mathrm{mol}}^{-1}$	[36]
Apparent charge trans. coef., ox	$\alpha_{\mathrm{ox}}^{v}$	1.5	-	2	-	chosen by [15]
Apparent charge trans. coef., red	$\alpha_{\mathrm{red}}^{v}$	1.5	-	2	-	chosen by [15]
Ionomer porosity	$\epsilon_{\mathrm{ion}}^{v}$	0.2	1	0.2	-	[37]
Ionomer tortuosity	$\tau_{\mathrm{ion}}^{v}$	2.236	1.5	2.2361	-	[18,37]
Mass transfer coef. O_2_	$k_{l,O2}$		0.0441		$m\;s^{-1}$	calc. in [15], data from [31]
Mass transfer coef. H_2_	$k_{l,H2}$		0.0992		$m\;s^{-1}$	calc. in [15], data from [31]
Sorption coef. vapor	$k_{g}^{\mathrm{sorp}}$		$5.7\times 10^{-6}$		$m\;s^{-1}$	[38]
Sorption coef. liquid	$k_{l}^{\mathrm{sorp}}$		$285\times 10^{-6}$		$m\;s^{-1}$	calc. with [39], data from [38]
Spec. ionomer surface	$a_{\mathrm{ion}}$		2.470		$m^{-1}$	calc. in [15]
Spec. pore surface	$a_{\mathrm{pore}}$		2.470		$m^{-1}$	assumed as $a_{\mathrm{ion}}$
Vapor diffusivity	$D_{\mathrm{vap}}$		$34.5\times 10^{-6}$		$m^{2}s^{-1}m^{2}/s$	[40]
CL Pore diameter	$d_{\mathrm{pore}}$		$0.1\times 10^{-6}$		m	[37]
Sherwood number	$Sh_{\mathrm{ec}}$		$2.04\times 10^{-3}$		-	[41]
Ref. O_2_ diffusivity	$D_{\mathrm{dsg},O2,0}$		$4.2\times 10^{-6}$		$m^{2}s^{-1}$	[31]
Ref. H_2_ diffusivity	$D_{\mathrm{dsg},H2,0}$		$4.9\times 10^{-6}$		$m^{2}s^{-1}$	[31]
Act. Energy O_2_ diff.	$E_{A,O2,0}$		18.38		$kJ\;{\mathrm{mol}}^{-1}$	[31]
Act. Energy H_2_ diff.	$E_{A,H2,0}$		16.51		$kJ\;{\mathrm{mol}}^{-1}$	[31]
Vap. enthalpy	$h^{\mathrm{Vap}}$		40.96		$kJ\;{\mathrm{mol}}^{-1}$	for $60\;{}^{\circ }C$ [15]
Entropy change	Δs		159.685		$J\;{\mathrm{mol}}^{-1}K^{-1}$	for $60\;{}^{\circ }C$ [15]

## Appendix B. Temperature Approximation

The temperature approximation is used to asset the low stoichiometry operation mode with regard to practical relevance. The heat sources are Joule heat $Q_{k}^{\mathrm{joule}}$ and heat by activation $Q_{k}^{\mathrm{act}}$. The heat sinks are latent heat demand for vaporization $Q_{k}^{\mathrm{vap}}$, and change in entropy $Q_{k}^{\mathrm{entr}}$:

(A40) $$\begin{array}{cc} Q_{k}^{\mathrm{joule}} & =i\left(z\right)\;\cdot \;\left(\Delta \varphi_{p,k}^{m}+\eta_{p,k}^{\mathrm{aCL}}+\eta_{p,k}^{m}+\eta_{e,k}^{\mathrm{aCL}}+\eta_{e,k}^{m}\right)\Delta z\;\cdot \;b^{\mathrm{aCL}} \end{array}$$

(A41) $$\begin{array}{cc} Q_{k}^{\mathrm{act}} & =i\left(z\right)\;\cdot \;\left(\eta_{\mathrm{act},k}^{\mathrm{aCL}}+\eta_{\mathrm{act},k}^{\mathrm{cCL}}\right)\Delta z\;\cdot \;b^{\mathrm{aCL}} \end{array}$$

(A42) $$\begin{array}{cc} Q_{k}^{\mathrm{vap}} & =h_{\mathrm{vap}}\;\cdot \;\left(j_{\mathrm{vap},k}^{\mathrm{aCL}}+j_{\mathrm{vap},k}^{\mathrm{cCL}}\right)\Delta z\;\cdot \;b^{\mathrm{aCL}} \end{array}$$

(A43) $$\begin{array}{cc} Q_{k}^{\mathrm{entr}} & =\frac{i(z)}{2F}\;\cdot \;T\Delta s\;\cdot \;\Delta z\;\cdot \;b^{\mathrm{aCL}} \end{array}$$

Here, $h_{\mathrm{vap}}$ is the specific vaporization enthalpy, and Δs is the entropy change. Both values are calculated for $T=60{}^{\circ }C$ with data from Trinke [15]. The heat is transported only by convective flux, which is calculated as follows:

(A44) $$Q_{k}^{\mathrm{conv}}=\sum_{j}^{v}\left(c_{p,j}\;\cdot \;g_{j,k}^{v}\;\cdot \;\Delta zb^{\mathrm{aCh}}\right)\Delta T\;:v=\mathrm{aCh},\mathrm{cCh},j=H_{2}O,H_{2},O_{2},\mathrm{vap}$$

with $c_{p,j}$, the molar heat capacity of each species calculated for $T=60{}^{\circ }C$ with the empircial equations for the gaseous phase:

(A45) $$\begin{array}{cc} c_{p,H2} & =29.09-0.8374\;\cdot \;\frac{T}{1000K}+2.013\;\cdot \;{\left(\frac{T}{1000K}\right)}^{2}\;\cdot \;J\;{\mathrm{mol}}^{-1}K^{-1} \end{array}$$

(A46) $$\begin{array}{cc} c_{p,O2} & =27.96+4.180\;\cdot \;\frac{T}{1000K}-0.1670\;\cdot \;{\left(\frac{T}{1000K}\right)}^{2}\;\cdot \;J\;{\mathrm{mol}}^{-1}K^{-1} \end{array}$$

(A47) $$\begin{array}{cc} c_{p,\mathrm{vap}} & =30.38+9.621\;\cdot \;\frac{T}{1000K}-1.185\;\cdot \;{\left(\frac{T}{1000K}\right)}^{2}\;\cdot \;J\;{\mathrm{mol}}^{-1}K^{-1} \end{array}$$

and $c_{p,H2O}=75.1J//K$ for liquid water [42]. When the sum of all produced heat sources is equal to the removed heat, the temperature increase ΔT can be roughly approximated.

Parameters

The parameters chosen for the model are listed in Table A3. Most parameters were chosen in accordance with the model from Trinke [15]. The model is setup qualitatively, so no parameters are fit to experimental results.

## Appendix C. Experimental Setup

In the following, the experimental cell is described briefly. A detailed view on the experimental setup can be found in our previous works [4,5].

The cell is set up as single channel PEMWE cell with an active area of $l^{\mathrm{aCL}}\;\cdot \;b^{\mathrm{aCL}}=50.4\;cm\times 0.45\;cm=22.68\;cm^{2}$. The channels are $b^{\mathrm{aCh}}/\mathrm{cCh}=1.5\;mm$ wide, $l^{\mathrm{aCh}}/\mathrm{cCh}=536\;mm$ long and $h^{\mathrm{aCh}}=2.0\;mm$ high in the anode and $h^{\mathrm{cCh}}=0.5\;mm$ high in the cathode. PTLs fabricated from titanium fibers on anode and cathode side (thickness: 1.0 mm) sandwich a commercial CCM. The aCL is built up from IrO_x_ catalyst, the cCL is a Pt/C catalyst both coated on a Nafion^®^117 membrane (thickness: ≈180 μm).

Along the cathode channel, 252 independent current density and temperature measurement plates are located, enabling a fine resolved CDD measurement each 2.0 mm. The anode channel and the PTLs are segmented into seven areas along the channel, with areas of identical size in the inlet, the middle, and the outlet region of the cell, which are used for local impedance spectroscopy. The power supply for the cell is either a *HEIDEN HE-LAB/SMS* power supply for potentiostatic operation, which can be replaced with a *Solartron ModuLab XM PSTAT* potentiostat, for local or integral impedance spectroscopy.

The cell has integrated heating and cooling elements along the channel enabling setting of constant temperature distributions along the channel. The cell is run on a lab-made test bench. The inlet water flow rate on the anode is controlled from 0.1 to $30\;g\;\min^{-1}$ with temperatures from 20 to $80{}^{\circ }C$ under ambient pressure on anode and cathode. Prior to each experiment, the cell is purged with nitrogen.

## Abbreviations

The following symbols, abbreviations and indexes are used in this manuscript:

**Latin Symbols**	
*a*	Volume specific surface, ($m^{-1}$)
$A_{\mathrm{geo}}$	Active geometrical cell area, ($m^{2}$)
$a_{\mathrm{vap}}$	Activity of vapor
*b*	Width of layer, (m)
*c*	Concentration, ($\mathrm{mol}m^{-3}$)
$c^{0}$	Reference concentration, ($\mathrm{mol}m^{-3}$)
$c_{p}$	Molar heat capacity, ($J\;{\mathrm{mol}}^{-1}K^{-1}$)
$d_{\mathrm{pore}}$	Pore diameter, (m)
*D*	Diffusion coefficient, ($m^{2}\;s^{-1}$)
*E*	Voltage, (V)
$E_{0}$	Reference Nernst potential, (V)
$E_{A}$	Activation energy, ($J\;{\mathrm{mol}}^{-1}K^{-1}$)
$E_{\mathrm{cell}}$	Cell voltage, (V)
EW	Equivalent weight of Nafion^®^, ($\mathrm{kg}\;{\mathrm{mol}}^{-1}$)
F	Faraday’s constant, ($96,485\;A\;s\;{\mathrm{mol}}^{-1}$)
*g*	Molar flux density in *z*-direction, ($\mathrm{mol}\;m^{-2}\;s^{-1}$)
$h_{\mathrm{vap}}$	Specific vaporization enthalpy, ($J\;{\mathrm{mol}}^{-1}$)
*i*	Current density, ($A\;m^{-2}$)
$i_{0}$	Exchange current density, ($A\;m^{-2}$)
*j*	Molar flux density in *x*-direction, ($\mathrm{mol}\;m^{-2}\;s^{-1}$)
*k*	Mass transfer coefficient, ($m\;s^{-1}$)
$\dot{m}$	Mass flow, ($\mathrm{kg}\;s^{-1}$)
$\tilde{M}$	Molar mass, ($\mathrm{kg}\;{\mathrm{mol}}^{-1}$)
$n_{\mathrm{drag}}$	Electro-osmotic drag coefficient
*p*	Pressure, (Pa)
*q*	Coupling exponent
*Q*	Heat flux, ($J\;s^{-1}$)
R	Universal gas constant, ($8.314\;J\;{\mathrm{mol}}^{-1}K^{-1}$)
Δs	Entropy change ($J\;{\mathrm{mol}}^{-1}K^{-1}$)
*S*	Solubility, ($\mathrm{mol}{\mathrm{Pa}}^{-1}m^{-3}$)
$Sh_{\mathrm{ec}}$	Electrochemical dimensionless Sherwood number
*T*	Temperature, (K)
*v*	Volume flux density, ($m\;s^{-1}$)
*x*	Sandwich coordinate, (m)
*z*	Channel coordinate, (m)
**Greek symbols**	
α	Apparent charge transfer coefficient
δ	Thickness, (m)
ϵ	Porosity
ζ	Dimensionless channel coordinate
η	Overpotential, (V)
κ	Conductivity, ($S\;m^{-1}$)
λ	Water content
$\lambda_{\mathrm{st}}$	Stoichiometric water ratio
ρ	Density, ($\mathrm{kg}\;m^{-3}$)
σ	Source term, ($A\;m^{-3}$) resp. ($\mathrm{mol}\;m^{-2}\;s^{-1}$)
τ	Tortuosity
φ	Potential, (V)
ω	Volume specific phase ratio
**Abbreviations**	
aCh	Anode channel
aCL	Anode catalyst layer
cCh	Cathode channel
cCL	Cathode catalyst layer
CCM	Catalyst coated membrane
CDD	Current density distribution
CL	Catalyst layer
LEL	Lower explosion limit
m	Membrane
PEM	Proton exchange membrane
PEMWE	Proton exchange membrane water electrolysis
PTL	Porous transport layer
**Sub- and superscripts**	
act	Activation
cons	Consumed
conv	Convective
dry	Dry
dsg	Dissolved gases
dsw	Dissolved water
e	Electron
eff	Effective
entr	Entropy
evo	Evolved
g	Gaseous water
H_2_	Hydrogen
*i*	Counter variable for membrane elements
ion	Ionomer
*j*	Placeholder variable for substances
joule	Joule heat
*k*	Counter variable in channel direction
l	Liquid water
L1, L2, L3, L4	Boundaries 1–4
L1−, L1+	Into boundary L1, out of boundary L1
*m*	Number of channel elements
*n*	Number of membrane elements
O_2_	Oxygen
ox	Oxidation
p	Proton
red	Reduction
ref	Reference
sat	Saturation
set	Set
sorp	Sorption
*v*	Placeholder variable for layers
vap	Vapor
w	Water
